# A small multigene hydroxyproline-*O*-galactosyltransferase family functions in arabinogalactan-protein glycosylation, growth and development in Arabidopsis

**DOI:** 10.1186/s12870-015-0670-7

**Published:** 2015-12-21

**Authors:** Debarati Basu, Lu Tian, Wuda Wang, Shauni Bobbs, Hayley Herock, Andrew Travers, Allan M. Showalter

**Affiliations:** Molecular and Cellular Biology Program, Department of Environmental and Plant Biology, Ohio University, Athens, OH 45701-2979 USA

**Keywords:** Arabidopsis, Arabinogalactan-proteins, AGP biosynthesis, Galactosyltransferase, *O-*glycosylation, Plant cell wall, Hydroxyproline, Galactose

## Abstract

**Background:**

Arabinogalactan-proteins (AGPs) are ubiquitous components of cell walls throughout the plant kingdom and are extensively post translationally modified by conversion of proline to hydroxyproline (Hyp) and by addition of arabinogalactan polysaccharides (AG) to Hyp residues. AGPs are implicated to function in various aspects of plant growth and development, but the functional contributions of AGP glycans remain to be elucidated. Hyp glycosylation is initiated by the action of a set of Hyp-*O*-galactosyltransferase (Hyp-*O*-GALT) enzymes that remain to be fully characterized.

**Results:**

Three members of the GT31 family (*GALT3*-*At3g06440*, *GALT4*-*At1g27120*, and *GALT6*-*At5g62620*) were identified as *Hyp-O-GALT* genes by heterologous expression in tobacco leaf epidermal cells and examined along with two previously characterized *Hyp-O-GALT* genes, *GALT2* and *GALT5*. Transcript profiling by real-time PCR of these five *Hyp-O-GALTs* revealed overlapping but distinct expression patterns. Transiently expressed GALT3, GALT4 and GALT6 fluorescent protein fusions were localized within Golgi vesicles. Biochemical analysis of knock-out mutants for the five *Hyp-O-GALT* genes revealed significant reductions in both AGP-specific Hyp-*O*-GALT activity and β-Gal-Yariv precipitable AGPs. Further phenotypic analysis of these mutants demonstrated reduced root hair growth, reduced seed coat mucilage, reduced seed set, and accelerated leaf senescence. The mutants also displayed several conditional phenotypes, including impaired root growth, and defective anisotropic growth of root tips under salt stress, as well as less sensitivity to the growth inhibitory effects of β-Gal-Yariv reagent in roots and pollen tubes.

**Conclusions:**

This study provides evidence that all five *Hyp-O-GALT* genes encode enzymes that catalyze the initial steps of AGP galactosylation and that AGP glycans play essential roles in both vegetative and reproductive plant growth.

**Electronic supplementary material:**

The online version of this article (doi:10.1186/s12870-015-0670-7) contains supplementary material, which is available to authorized users.

## Background

Arabinogalactan-proteins (AGPs) are members of the hydroxyproline (Hyp)-rich cell wall glycoprotein superfamily and are hyperglycosylated by *O*-linked AG polysaccharides. AGPs are found in cell walls, plasma membranes, and extracellular secretions of virtually all plant cells, tissues and organ types [1]. Moderately sized gene families encode a variety of AGP protein backbones throughout the plant kingdom. For example, based on bioinformatics studies, Arabidopsis contains 85 AGP genes, while rice contains 69 AGP genes [2, 3]. Moreover, these genes are spatially and temporally expressed in a variety of patterns, which likely relates to their multiple functions.

AGPs are implicated to function in various aspects of plant growth and development, including root elongation, somatic embryogenesis, hormone responses, xylem differentiation, pollen tube growth and guidance, programmed cell death, cell expansion, salt tolerance, host-pathogen interactions, and cellular signaling [4–10]. However, there remains a lack of understanding of the biophysical and biochemical modes of action of any individual AGP. This lack of understanding regarding function also extends to the carbohydrate moieties or AG polysaccharides, which extensively decorate AGP core proteins and largely define their interactive surfaces.

Given the importance of understanding plant cell wall biosynthesis particularly with respect to biofuel production, much of the recent work on AGPs has focused on their biosynthesis. Such efforts have identified several of the biosynthetic glycosyltransferase (GT) genes/enzymes responsible for AG polysaccharide production [6, 11]. In particular, the following enzymes were identified and cloned: two α-1,2-fucosyltransferases (FUT4 and FUT6) which are members of the CAZy GT-37 family [12–14], two hydroxyproline-*O*-galactosyltransferases (GALT2 and GALT5) which are members of GT-31 and contain a galectin domain [15, 16], three other hydroxyproline-*O*-galactosyltransferases (HPGT1-HPGT3) which are members of GT-31 but lack a galectin domain [17], one β-1,3-galactosyltransferase (At1g77810) which is a member of GT-31 [18], one β-1,6-galactosyltransferase with elongation activity which is a member of GT-31 (GALT31A) [19], one β-1,6-galactosyltransferase with branch initiation and branch elongating activities which is a member of GT-29 (GALT29A) [20]*,* three β-1,6-gluronosyltransferases which are members of GT-14 (GlcAT14A, GlcAT14B, GlcAT14C) [21, 22], and a putative AGP β-arabinosyltransferase (RAY1) which is a member of the GT-77 family [23].

The hydroxyproline-*O*-galactosyltransferases (Hyp-*O*-GALT) that add galactose onto the peptidyl Hyp residues in AGP core proteins represent the first committed step in AG polysaccharide addition and represent an ideal control point to investigate the contribution of AG polysaccharides to AGP function. Previously, we demonstrated that *GALT2* (*At4g21060*) and *GALT5* (*At1g74800*) are members of a small multigene family and encode Hyp-GALTs [15, 16]. In addition, extensive phenotypic characterization of allelic *galt2* and *galt5* single mutants and *galt2galt5* double mutants at the biochemical and physiological levels was performed which corroborated the roles of these two enzymes in AG biosynthesis and elucidated the contributions of AG polysaccharides to AGP function. Here, we extend that work by characterizing the remaining GALT members (i.e., GALT1, GALT3, GALT4, and GALT6) of this small six-membered gene family, which are distinguished by encoding a GALT domain as well as a GALECTIN domain.

## Results

### *In silico* analysis of GALT1, GALT3, GALT4, and GALT6

This study focused on the six-member gene/protein family in Arabidopsis, which is found within the CAZy GT31 family and distinguished by the presence of both a GALT (pfam 01762) and a GALECTIN (pfam 00337) domain. Recently, two of these six members, GALT2 (At4g21060) and GALT5 (At1g74800) were demonstrated to catalyze the addition of galactose onto Hyp residues of AGP backbones [15, 16]. Another member of this family, GALT1, encoded by *At1g26810*, was previously characterized and identified as a β–1,3-galactosyltransferase involved in the formation of the Lewis a epitope on *N*–linked glycans [24]. The open reading frames of the remaining members, *At3g06440* (*GALT3*), *At1g27120* (*GALT4*), and *At5g62620* (*GALT6*) correspond to 1860, 2022 and 2046 bp and specify proteins with 619 (70 kDa), 673 (77.0 kDa), and 681 (77.7 kDa) amino acids, respectively (Additional file 1: Table S1). The six proteins share amino acid identities ranging from 35 to 70 % (Additional file 1: Table S2). In addition, comparisons of these six members were performed with the three recently identified AGP-specific Hyp-*O*-GALTs (HPGT1, HPGT2, and HPGT3), which are also within the GT31 family and contain a GALT domain but lack a GALECTIN domain [17]. All nine proteins were predicted to be type II Golgi localized integral membrane proteins by several subcellular localization prediction programs (TargetP, http://www.cbs.dtu.dk/services/TargetP/ and Golgi Predictor, http://ccb.imb.uq.edu.au/golgi/) [25], Additional file 1: Table S2). These nine GALTs were also submitted the TMHMM server (http://www.cbs.dtu.dk/services/TMHMM/) for prediction of transmembrane domains (TMDs), a typical type II membrane topology commonly found in GTs [26] (Additional file 1: Figure S1). All were predicted to have a single TMD except for GALT3, HPGT2, and HPGT3, which instead contained hydrophobic regions that may serve as an anchor to the Golgi membrane. Hydrophobic cluster analysis (HCA) was performed by submitting the protein sequences to the drawhca server (http://bioserv.impmc.jussieu.fr/hca-form.html) and used to identify the hydrophobic pockets containing the “DXD” motifs of the six GALTs; this analysis also included two previously characterized AGP-related GT31 members, GALT31A and At1g77810, which are involved with the elongation of β-1,6-galactan side chains and the β-1,3 backbone of AG polysaccharides, respectively (Additional file 1: Figure S2) [18, 19, 27, 28]. HCA analysis revealed conserved DDD motifs in all the proteins contained within various hydrophobic pockets. The DXD motif is implicated in the binding the divalent metal ion that assists in anchoring the pyrophosphoryl group of the UDP-sugar donor in the enzyme’s active site [18]. Co-expression analysis was performed using GENEMANIA (http://www.genemania.org/) and revealed that *GALT3*, *GALT4*, and *GALT6* expression is tightly correlated with well-characterized AGP-specific GT31 members as well as with a number of AGPs (Additional file 1: Table S3) [15, 18, 19, 24, 29].

### Transiently expressed *GALT* genes in *Nicotiana* have AGP-specific Hyp-*O*-GALT activity

**Fig. 1:**
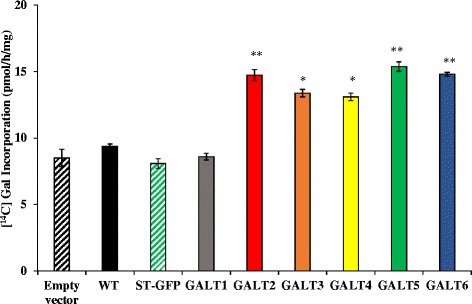
Hyp-*O*-GALT activity of GALT1-GALT6 transiently expressed in *N. tabacum*. GALT1-GALT6 were expressed in epidermal cells of tobacco leaves by *Agrobacterium*-mediated transient expression, which were used for the preparation of Golgi-enriched microsomal membrane proteins for the Hyp-GALT assays. Synthetic peptide [AO]_7_ was used as substrate acceptor. WT tobacco leaves infiltrated with *Agrobacterium* GV3101 strain (Empty vector), WT tobacco leaves, and WT tobacco leaves infiltrated with ST fused with GFP were used as controls. Experiments were performed using duplicate samples and data represent the mean ± SD from two independent experiments. Asterisks indicate mean values significantly different from the WT (Dunnett’s test, *P <0.05; **P <0.01)

For biochemical characterization, full-length *GALT1*, *GALT2*, *GALT3*, *GALT4, GALT5,* and *GALT6* gene constructions, each harboring an N-terminal 6XHis tag, were transiently expressed in the leaves of *Nicotiana tabacum*. Leaves infiltrated with desired constructs were initially separated into three fractions: supernatant, total microsomal membranes and Golgi-enriched microsomal membranes. The highest GALT activity was observed in Golgi-enriched detergent permeablized microsomal membranes (Additional file 1: Table S4), and thus this fraction was subsequently used as the enzyme source in transient assays (Fig. 1). Here, five of the six GALTs (i.e., GALT2-GALT6) displayed Hyp-*O*-GALT activity, when compared to controls [tobacco WT leaves alone or infiltrated with either an empty vector or an unrelated glycosyltransferase gene, sialyl transferase (ST)]. Previously characterized GALT2 and GALT5 were used as positive controls for this assay, while GALT1 effectively served as a negative control, given its involvement with *N*-glycan biosynthesis [15, 16, 24].

### Substrate specificities of GALT2-GALT6

**Fig. 2:**
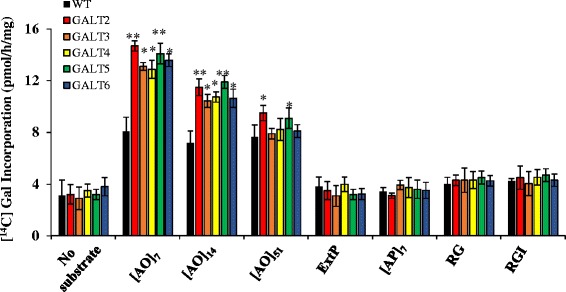
Substrate specificity of transiently expressed GALT2-6. Detergent permeablized tobacco microsomal membranes obtained from transiently expressed GALT2-6 served as the enzyme source in the GALT reactions. Various peptide and polysaccharide acceptor substrates were tested including: 1) [AO]_7_, [AO]_14_, and d[AO]_51_ which contain 7, 14, and 51 [AO] units, respectively, 2) a chemically synthesized extensin peptide (ExtP) containing repetitive SO_4_ units, 3) [AP]_7_ which contains 7 [AP] units, 4) Rhamnogalactan I (RGI) from potato pectin, and 5) RG from soybean pectin. Microsomes obtained from WT tobacco leaves infiltrated with empty pMDC32 vector were used as a negative control and depicted as WT. Enzyme reactions were done in triplicate and mean values ± SE are presented. Asterisks indicate values significantly different from the WT (Dunnett’s test, *P <0.05; **P <0.01)

Various potential substrate acceptors were tested to investigate enzyme specificity of GALT3, GALT4, and GALT6. Namely, [AO]_7_, [AO]_14_, and d[AO]_51_, consisting of non-contiguous peptidyl Hyp residues, were used to examine the effect of these model AGP peptide sequences of various lengths on GALT activity. [AP]_7_, consisting of alternating Ala and Pro residues, was tested for the requirement of peptidyl Hyp for galactosylation. ExtP, a chemically synthesized extensin peptide consisting of contiguous peptidyl Hyp residues, tested whether contiguous peptidyl Hyp residues act as potential acceptors. Two commercially available pectic polysaccharides, Rhamnogalactan I from potato and Rhamnogalactan (a mixture of RGI and RGII) from soybean, were also tested as potential substrates acceptors. All the non–AGP substrate acceptors, including [AP]_7_, failed to incorporate [^14^C]Gal, indicating the GALT activity was specific for AGP sequences containing non-contiguous peptidyl Hyp (Fig. 2). It is interesting to note that GALT2 and GALT5 expressed in tobacco displayed higher activity than when expressed in *Pichia*, even after taking into account the relatively high background activity in tobacco. This indicates that there are plant-specific factors or accessory proteins critical for Hyp-*O*-GALT activity [15, 16].

### Additional biochemical characterization of Hyp-*O*-GALTs

Heterologously expressed Hyp-*O*-GALTs required the divalent cation Mn^+2^ for maximal activity and utilized UDP- galactose solely as the sugar donor (Additional file 1: Figure S3). This is in contrast to GALT2 and GALT5 expressed in *Pichia*, which required Mg^+2^ for its optimal activity [15, 16].

### Expression profiles of the *Hyp-O-GALT* genes

**Fig. 3:**
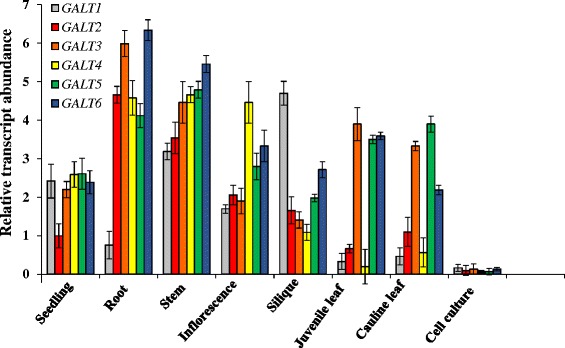
Expression patterns of the six membered *GALT* gene family. qPCR analysis of *GALT1*-*GALT6* expression Arabidopsis organs and cell cultures. Roots were obtained from 14 day old seedlings grown on MS plates with 1 % sucrose and a week old cell suspension culture was used for RNA extraction. The level of expression was calculated relative to the *UBQ10* gene (mean ± SE of three biological replicates)

qRT-PCR and data mining of public databases were used to analyze expression profiles of the *Hyp-O-GALT* genes. qRT-PCR analysis indicated that *GALT1*-*GALT6* are broadly expressed and have overlapping but distinct expression patterns (Fig. 3). These Q-PCR data were in good agreement with public expression data available from GENEVESTIGATOR and the eFP browser [30, 31] as well as from the previous study by Strasser et al. [24] (Additional file 1: Figure S4). Data from large-scale transcriptomic databases were used to provide insight into *GALT* expression and provide clues as to where to focus phenotypic analysis of *GALT* knockout mutant plants. Notable patterns of expression were as follows: highest expression of *GALT6* was observed in senescent leaves followed by seed, seed coat, root hairs, flowers, and siliques, whereas *GALT4* was predominantly expressed in young flowers, mature flowers with siliques and mature siliques. *GALT3* was abundant in roots, mature pollen, and hypocotyl (Additional file 1: Figure S4).

Numerous studies indicate that pollen tubes undergo dramatic transformations while growing in the pistil, where they rapidly grow, perceive and respond to navigational cues secreted by the pistil, with AGPs playing a critical role in such interactions [32–34]. Nonetheless, genes expressed by pollen tubes in response to growth in the pistil are poorly characterized. Qin et al. [35] utilized the novel combination of semi in vitro pollination followed by microarray analysis to identify genes specifically involved in pollen-pistil interaction, including the *Hyp-O-GALTs. GALT5* had the highest expression followed by *GALT2* and *GALT4*, whereas *HPGT3* was only expressed in later stages of pollen elongation. Furthermore, it is interesting to note that there was a temporal difference in the expression patterns of these *Hyp-O-GALTs* during pollen elongation (Additional file 1: Figure S4).

In addition, transcriptome analyses using RNA extracted from laser-capture dissected seed coat tissue (http://seedgenenetwork.net/arabidopsis) indicated that all five *Hyp-O-GALT* transcript levels displayed unique expression patterns in the seed coat during embryogenesis (Additional file 1: Figure S5) [36]. Notably, *GALT6* was expressed throughout seed development, while expression of *GALT2* and *GALT5* transcripts was higher in the early stages compared to the later stages of seed development. In contrast, *GALT4* was only observed at later stages of seed development, while *GALT3* showed the least expression in seeds.

### GALT3, GALT4, and GALT6 are targeted to Golgi vesicles

**Fig. 4:**
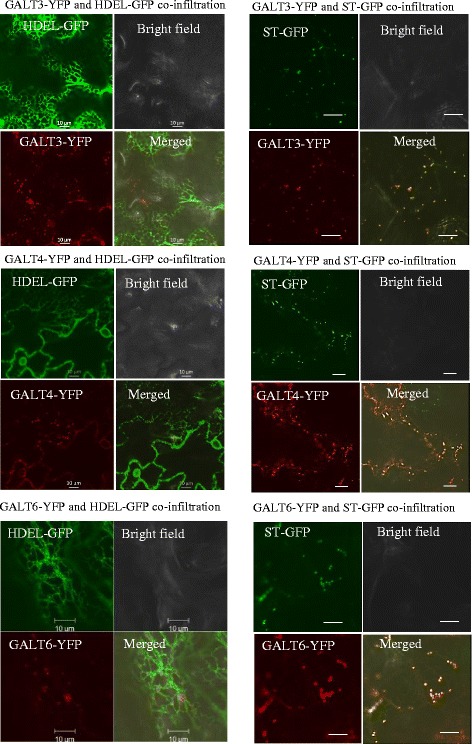
Subcellular localization of transiently expressed GALT3-YFP, GALT4-YFP, and GALT6-YFP in *N. tabacum*. GALT3-YFP, GALT4-YFP, and GALT6-YFP fusion constructions were expressed under the control of the CaMV 35S promoter in *N. tabacum*. Transiently expressed GALT3-YFP, GALT4-YFP, and GALT6-YFP co-localized with sialyl transferase (ST)-GFP fusion protein (a Golgi marker), but not with HDEL-GFP fusion protein (an ER marker). These constructs were examined by laser-scanning confocal microscopy under fluorescent and white light, and the fluorescent images were merged to observe co-localization. Size bar = 10 μm

Transient expression of C-terminal YFP fusions to GALT3, GALT4, and GALT6 were infiltrated in *N. tobaccum* epidermal leaf cells to examine the subcellular localization of these enzymes (Fig. 4). Overlays of GALT3-YFP, GALT4-YFP, and GALT6-YFP individually co-expressed with the Golgi marker protein, sialyl transferase, fused to GFP (ST-GFP) indicated that all three GALTs were localized to the Golgi apparatus. Furthermore, the possibility that they were localized in the ER was excluded, as the GALT-YFP fusion constructions were not co-localized with the ER marker, HDEL fused with GFP (HDEL-GFP). Singly infiltrated controls for ST-GFP, HDEL-GFP, and GALT-YFP were analyzed to optimize gain and pinhole settings for each channel and to exclude any bleed through fluorescence between channels (Additional file 1: Figure S6).

### *GALT3*, *GALT4,* and *GALT6* mutants show AGP biochemical defects

**Fig. 5:**
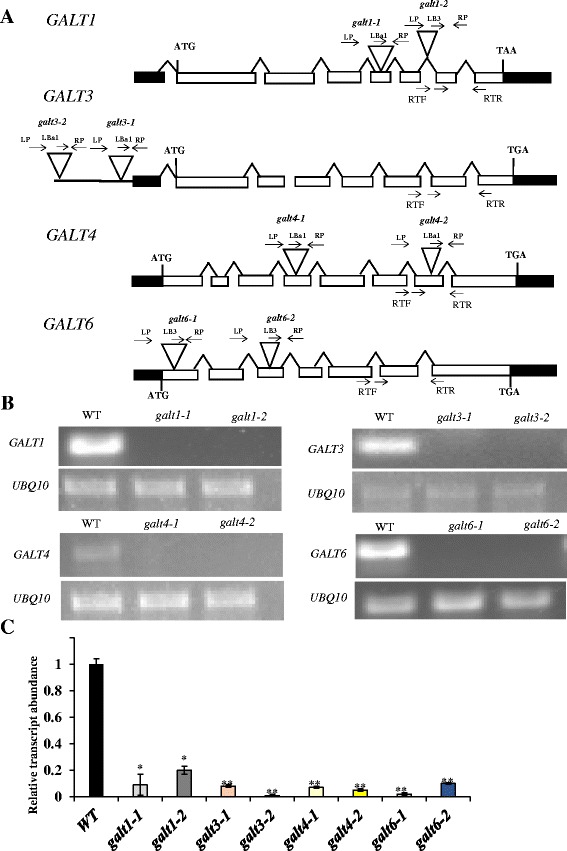
Schematic gene models, locations of T-DNA mutant insertions, and transcript analysis of *GALT1*, *GALT3*, *GALT4*, and *GALT6*. **a** *GALT1, GALT3. GALT4* and *GALT6* gene structures and T-DNA insertion sites in *galt1-1*, *galt1-2*, *galt3*-*1, galt3-2, galt4-1, galt4-2, galt6-1,* and *galt6-2* mutants. The intron-exon structures of *GALT1, GALT3, GALT4,* and *GALT6* are indicated (introns are drawn as lines and exons as *rectangles*, with *white rectangles* representing coding sequences and *black rectangles* representing UTRs). Sites of T-DNA insertions are marked (*triangles*) as are the locations of primer sequences (*arrows* above the genes) used for PCR screening. **b** RT-PCR analysis of transcripts from rosette leaves of 14-day-old WT (Col-0) and allelic homozygous *galt1, galt3*, *galt4* and *galt6* mutant lines. Arrows below the genes in (**a**) indicate the position of primers (denoted as RTF and RTR) used for RT-PCR analysis of transcript levels. *UBQ10* primers were used as internal controls. **c** Quantitative real-time reverse transcription -PCR (qRT-PCR) analysis was performed to quantify and compare transcript levels of the indicated genes with that of corresponding WT gene. In other words, the relative expression level of the *GALT* genes in the mutants was compared to WT values, which were set to a value of 1.0 for each of the *GALT* genes. Asterisks indicate values significantly different from the WT expression of the indicated genes (Dunnett’s test, *P <0.01; **P <0.001)

Two independent allelic mutant lines with T-DNA insertions were identified for each of the six *GALT* genes in order to examine the biochemical roles of the Hyp-*O*-GALTs in vivo. Homozygous mutants were generated, identified by PCR, and confirmed by sequencing (Fig. 5a). RT-PCR and qRT-PCR analysis showed that virtually no transcripts could be detected in the mutants (Fig. 5b and c). Significant reductions in GALT activity as well as β-Gal-Yariv precipitable AGPs obtained from 14-d old seedlings were observed in knock-out mutants of *GALT3* (*galt3-1* and *galt3-2*), *GALT4* (*galt4-1* and *galt4-2*), and *GALT6* (*galt6-1* and *galt6-2*) compared to WT (Table 1). Such reductions were previously reported for knock-out mutants of *GALT2* (*galt2-1* and *galt2-2*), *GALT5* (*galt5-1* and *galt5-2*), and a *galt2galt5* double mutant and were used here as positive controls [16]. Consistent with the findings that GALT1 synthesizes Lewis a structures and lacks Hyp-*O*-GALT activity (Fig. 1), knock-out mutants of *GALT1* (*galt1-1* and *galt1-2*) demonstrated no such reductions and were indistinguishable from WT (Table 1) [24].

**Table 1:** GALT activity and amount of β-Gal-Yariv precipitated AGPs in WT and *galt* mutants

Genotype	GALT activity (pmol/h/mg)	β-Gal-Yariv precipitated AGPs (μg/g)
WT	7.10 ± 0.90	13.60 ± 0.75
*galt1-1*	6.80 ± 0.37	13.30 ± 0.95
*galt1-2*	7.20 ± 0.65	13.30 ± 0.95
*galt2-1*	5.53 ± 1.20^b^	9.91 ± 2.80^b^
*galt2-2*	5.90 ± 1.01^b^	9.28 ± 1.50^b^
*galt3-1*	6.08 ± 1.20^a^	12.10 ± 0.95
*galt3-2*	5.51 ± 1.01^b^	12.30 ± 0.80^a^
*galt4*-*1*	6.04 ± 2.20^a^	12.00 ± 1.10^a^
*galt4*-*2*	5.83 ± 1.50^b^	11.90 ± 1.20^a^
*galt5-1*	5.45 ± 1.10^b^	7.90 ± 2.10^b^
*galt5-2*	4.90 ± 1.50^b^	8.10 ± 1.20^b^
*galt6-1*	5.30 ± 0.44^b^	10.90 ± 0.59^a^
*galt6-2*	5.00 ± 1.71^b^	10.30 ± 1.54^a^
*galt2galt5*	4.64 ± 0.54^b^	5.63 ± 0.39^b^

Detergent-solubilized microsomal fractions were used for performing a standard Hyp-GALT assay, and AGPs were extracted, precipitated by β-Gal-Yariv reagent, and quantified from 14-day-old plants. The values are averages of at least two independent experiments from two biological replicates. Letters ‘a’ and ‘b’ denote a significant difference from the wild type (Dunnett’s test, *P* <0.05; *P* <0.01) respectively

Given the differential expression of these Hyp-*O*-GALTs and the broad expression of AGPs, AGPs were also quantified from other organs in the mutants. Similar patterns of reductions in β-Gal-Yariv precipitable AGPs were observed in these other organs for these mutants. In general, disruption of any of the five *GALTs* (*GALT2-GALT6*) caused a significant reduction in AGP content, with most significant effects being exhibited by *galt5* in stems, *galt4* in siliques, and *galt6* in senescent leaves (Table 2). These data on AGP quantification in the mutants were consistent with the expression profile data for *GALT2-GALT6*. Profiles of these β-Gal-Yariv precipitable AGPs produced by RP-HPLC were also examined for various *galt* mutants and revealed that virtually all these AGPs, as opposed to a single or subset of these AGPs, were affected when compared to WT or *galt1* control profiles (Additional file 1: Figure S7). Furthermore, the AGP peaks in the *galt3, galt4,* and *galt6* mutants eluted later and thus had less glycosylated protein compared to the WT or *galt1* control AGP peaks, consistent with reduced Hyp-galactosylation.

**Table 2:** Amount of β-Gal-Yariv precipitated AGPs in WT and *galt* mutants

Genotype	Stem	Silique	Flower	Senescent leaves
WT	35.7 ± 3.4	15.5 ± 2.5	17.0 ± 0.4	28.4 ± 3.6
*galt1-1*	34.9 ± 2.1	15.2 ± 1.8	16.5 ± 0.5	28.5 ± 2.4
*galt1-2*	35.1 ± 2.0	15.8 ± 2.3	17.2 ± 0.9	28.1 ± 3.5
*galt2-1*	25.3 ± 3.3^a^	11.8 ± 1.3^a^	13.9 ± 0.5^b^	24.4 ± 3.1^a^
*galt2-2*	26.1 ± 2.7^a^	11.5 ± 1.7^a^	13.4 ± 0.4^b^	24.0 ± 2.6^a^
*galt3-1*	29.2 ± 4.1	12.5 ± 0.6^a^	15.7 ± 0.7^a^	26.9 ± 3.8^a^
*galt3-2*	28.6 ± 2.9	11.9 ± 1.2^a^	15.0 ± 0.2^a^	27.0 ± 2.2
*galt4-1*	29.7 ± 3.7	9.9 ± 0.8^b^	11.9 ± 0.1^b^	27.1 ± 2.4
*galt4-2*	29.5 ± 1.5	8.3 ± 0.5^b^	12.1 ± 0.4^b^	27.5 ± 3.1
*galt5-1*	23.6 ± 3.3^b^	10.7 ± 0.9^b^	12.7 ± 0.9^b^	25.1 ± 3.5^a^
*galt5-2*	23.7 ± 2.8^b^	11.1 ± 0.4^a^	12.9 ± 0.8^b^	24.9 ± 4.6^a^
*galt6-1*	27.3 ± 2.3^a^	10.4 ± 0.7^b^	12.2 ± 0.4^b^	23.0 ± 3.7^b^
*galt6-2*	26.9 ± 3.6^a^	11.2 ± 0.8^a^	12.4 ± 0.7^b^	22.0 ± 2.9^b^
*galt2galt5*	25.3 ± 2.4^a^	11.0 ± 0.9^a^	12.3 ± 0.5^b^	24.5 ± 3.1^a^

Letters ‘a’ and ‘b’ denote a significant difference from the wild type (Dunnett’s test, *P* <0.05; *P* <0.01; respectively). Stem, silique, and flowers were obtained from 30-day-old plants, whereas senescent leaves were obtained from 45-day-old plants

### *GALT3*, *GALT4,* and *GALT6* mutants exhibit root hair defects

**Fig. 6:**
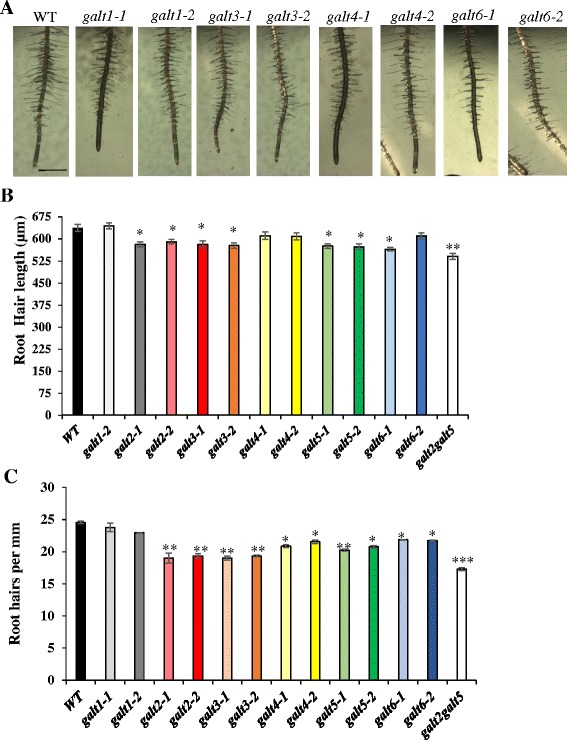
Root hair length and density reduced in the *galt3, galt4, and galt6* mutants. **a** WT, *galt1*, *galt3, galt4*, and *galt6* plants were grown on MS agar plates for 10 days. Bar = 1 mm. **b** Quantification of root hair length and **c** root hair density of the *galt* mutants. Asterisks indicate significantly reduced root hair length and density compared with WT controls according to Dunnett’s test (*P <0.05; **P <0.01; *n* >300)

To investigate the physiological function of these six GALTs in vivo, mutants were grown on MS plates and compared to WT. No significant phenotypic differences in primary root growth were observed with the exception of the root hairs. Single mutant knock-out lines for *GALT3*, as well as for *GALT2* and *GALT5* and the *galt2galt5* double mutant, consistently displayed shorter and less dense root hairs compared to WT; knock-out lines for *GALT6*, *GALT4,* and *GALT1* displayed either less severe or no such root hair phenotypes (Fig. 6).

### *GALT4* and *GALT6* mutants display reduced seed set

**Fig. 7:**
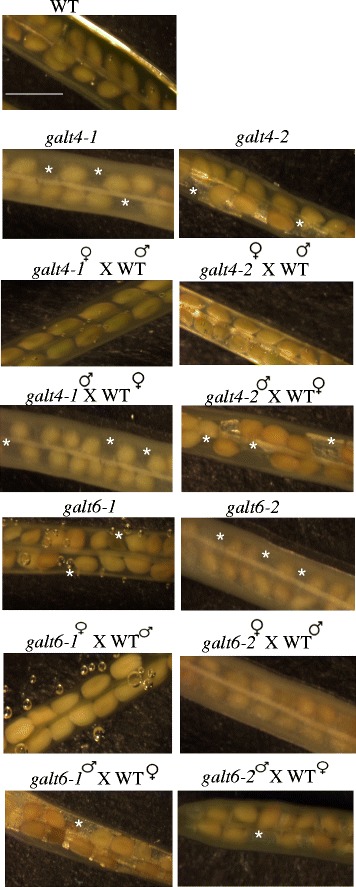
Silique morphology of *galt4* and *galt6* mutant plants along with reciprocal crosses of these mutants to WT plants. Siliques were treated with ethanol to allow for easy observation of the seeds. Absence of ovules is indicated with an asterisk. Bar = 100 μm

The *galt4* and *galt6* mutants displayed a 16 and 13 % reduction in seed set, respectively (Fig. 7 and Table 3). Reciprocal crosses of the *galt4* and *galt6* mutants to wild type plants were performed to determine whether this defect was conferred by the male or female gametophyte. Such crosses indicated that the male gametophyte of these mutants was mainly responsible for conferring reduced seed set (Fig. 7 and Table 3). Pollen were consequently examined with Alexander’s stain which indicated that pollen were viable (Additional file 1: Figure S8A). Furthermore, in vitro pollen germination did not exhibit altered germination frequency in *galt4* and *galt6* mutants compared to WT (Additional file 1: Figure S8B and S8C).

**Table 3:** Weight, length, and seed number from WT and *galt* siliques

Genotype	Silique length (mm)	Seeds/Silique	Seed weight (mg)
WT ♀ × WT♂	12.90 ± 0.87	56.10 ± 3.80	4.50 ± 0.34
*galt1-2*♀ × *galt1-2*♂	13.04 ± 0.84	54.87 ± 2.70	4.65 ± 0.28
*galt2-1*♀ × *galt2-1*♂	12.51 ± 0.22	52.45 ± 3.52	4.20 ± 0.91
*galt2-2*♀ × *galt2-2*♂	13.21 ± 0.34	53.65 ± 2.93	4.65 ± 0.44
*galt3-1*♀ × *galt3-1*♂	13.06 ± 0.68	52.12 ± 3.29	4.63 ± 0.34
*galt3-2*♀ × *galt3-2*♂	12.80 ± 0.77	53.37 ± 2.66	4.50 ± 0.37
*galt4-1*♀ × *galt4-1*♂	13.06 ± 0.56	47.37 ± 2.28^b^	3.26 ± 0.40^a^
*galt4-2*♀ × *galt4-2*♂	12.85 ± 0.59	47.50 ± 2.44^b^	3.41 ± 0.32^a^
*galt5-1*♀ × *galt5-1*♂	13.32 ± 0.34	53.67 ± 3.4	4.23 ± 0.54
*galt5-2*♀ × *galt5-2*♂	13.65 ± 0.89	55.28 ± 2.7	4.67 ± 0.89
*galt6-1*♀ × *galt6-1*♂	13.10 ± 0.57	49.11 ± 4.24^b^	3.41 ± 0.18^a^
*galt6-2*♀ × *galt6-2*♂	13.60 ± 0.56	50.56 ± 2.79^b^	3.72 ± 0.27^a^
WT♀ × *galt4-1* ♂	13.10 ± 0.73	45.10 ± 6.40^b^	3.70 ± 0.56^a^
WT♀ × *galt4-2*♂	12.91 ± 0.45	43.45 ± 4.90^b^	3.54 ± 0.38^a^
*galt4-1*♀ × WT♂	13.06 ± 0.56	53.37 ± 4.28	4.56 ± 0.40
*galt4-2*♀ × WT♂	12.85 ± 0.59	52.10 ± 1.40	4.34 ± 0.62
WT♀ × *galt6-1* ♂	13.00 ± 0.54	49.70 ± 7.40^b^	3.50 ± 0.56^a^
WT♀ × *galt6-2* ♂	12.88 ± 0.47	50.60 ± 4.40^b^	3.50 ± 0.56^a^
*galt6-1*♀ × WT♂	13.06 ± 0.71	53.37 ± 4.28	4.56 ± 0.40
*galt6-2*♀ × WT♂	13.13 ± 0.96	53.37 ± 4.28	4.56 ± 0.40

Siliques were obtained from 6-week-old plants (*n* = 20). Letters ‘a’ and ‘b’ denote a significant difference from the wild type (Dunnett’s test, *P* <0.05; *P* <0.01 respectively)

### *GALT3* and *GALT6* mutants demonstrate reduced staining of adherent seed coat mucilage

**Fig. 8:**
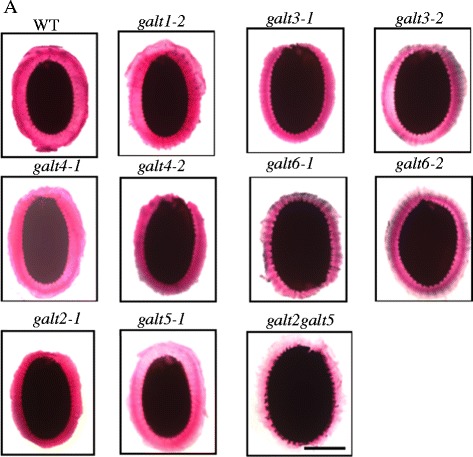
Pectin staining of seed coat mucilage in wild type, *galt1-galt6* single mutants, and *galt2galt5* double mutants. Seeds of the indicated genotypes were prehydrated with water for 90 min and stained with ruthenium red to visualize pectin using a Nikon Phot-lab2 microscope coupled with a SPOT RT color CCD camera and SPOT 4.2 analysis software. Bar = 100 μm

Prior evidence for the involvement of AGPs (SOS5) and GALT2/GALT5 in seed coat mucilage prompted an examination of the potential functions of *GALT3*, *GALT4,* and *GALT6* in modifying seed coat mucilage [16, 37, 38]. The effect of disruption of the six *GALT* gene family members on adherent seed mucilage was investigated by staining hydrated seeds with ruthenium red, which stains negatively charged biopolymers such as pectin [39]. The *galt3-1, galt3-2, galt6-1, galt6-2,* and *galt2galt5* mutant seeds showed a thin staining pattern of the adherent mucilage, whereas WT, *galt2*, *galt4, galt5*, and *galt1* seeds showed an intense, regular, spherical staining pattern (Fig. 8). In addition, adherent mucilage mass and volume were measured to confirm the reduction of adherent mucilage thickness. No difference was observed in adherent mucilage mass between WT and *galt* single and double mutant seeds, whereas the adherent mucilage size of *galt3*, *galt6,* and *galt2galt5* was substantially reduced (20 ~ 30 %) compared with WT (Table 4). In contrast, *galt1*, *galt4*, *galt2,* and *galt5* mutants were less dramatically altered (5 ~ 13 %) compared to WT.

**Table 4:** Determination of adherent mucilage mass and size in WT and *galt* mutants

Genotype	Mass (μg)	Size (mm^3^)
WT	1.82 ± 0.05	0.47 ± 0.13
*galt1-2*	1.76 ± 0.06	0.45 ± 0.03
*galt2-1*	1.80 ± 0.09	0.44 ± 0.04^a^
*galt2-2*	1.77 ± 0.06	0.45 ± 0.07^a^
*galt3-1*	1.80 ± 0.09	0.37 ± 0.08^b^
*galt3-2*	1.75 ± 0.08	0.35 ± 0.05^b^
*galt4*-*1*	1.79 ± 0.05	0.42 ± 0.10^a^
*galt4*-*2*	1.81 ± 0.05	0.39 ± 0.20^a^
*galt5-1*	1.80 ± 0.05	0.41 ± 0.30^a^
*galt5*-*2*	1.81 ± 0.05	0.39 ± 0.25^b^
*galt6-1*	1.83 ± 0.04	0.35 ± 0.04^b^
*galt6-2*	1.77 ± 0.07	0.37 ± 0.05^b^
*galt2galt5*	1.75 ± 0.09	0.30 ± 0.05^b^

The mass and size values are the average mass and size of adherent mucilage of 100 seeds of triplicate assays ± SE. Letters ‘a’ and ‘b’ denote a significantly difference from the wild type (Dunnett’s test, *P* <0.05; *P* <0.01 respectively)

In order to confirm and quantify the changes in non-adherent (soluble) and adherent mucilage, WT and *galt* mutant seeds were analyzed. Sequential extraction of seeds with ammonium oxalate, 0.2 N NaOH, and 2 N NaOH was performed to assess changes in the soluble and adherent mucilage (Table 5). Both *galt6* and *galt3* seeds had a significant increase in the total sugar present in the ammonium oxalate and 0.2 N NaOH extracts (soluble and weakly attached pectins) compared to wild type seeds (or *galt1* mutants). Less significant differences were observed in *galt2*, *galt4*, and *galt5* single mutants, whereas more significant differences were observed in *galt2galt5* mutants. All the *galt* mutants except for *galt1* displayed a decrease in total sugars in the 2 N NaOH extracts, which represent the majority of the adherent mucilage and contain strongly linked pectins and cross-linking glycans/hemicelluloses [40, 41].

**Table 5:** Quantification of total sugars from WT and *galt* mucilage sequentially extracted using ammonium oxalate, 0.2 N NaOH, and 2 N NaOH

	Extract^a^		
Genotype	Ammonium oxalate	0.2 N NaOH	2 N NaOH
WT	0.85 ± 0.05	1.04 ± 0.03	0.81 ± 0.04
*galt1-2*	0.83 ± 0.07	1.05 ± 0.05	0.84 ± 0.03
*galt2-1*	0.95 ± 0.03^b^	1.12 ± 0.04^b^	0.61 ± 0.05^b^
*galt2-2*	0.97 ± 0.70^b^	1.19 ± 0.03^b^	0.54 ± 0.06^b^
*galt3-1*	1.30 ± 0.09^c^	1.29 ± 0.05^c^	0.53 ± 0.05^b^
*galt3-2*	1.28 ± 0.05^c^	1.27 ± 0.07^c^	0.52 ± 0.07^b^
*galt4-1*	0.88 ± 0.20	1.09 ± 0.02	0.79 ± 0.01
*galt4-2*	0.90 ± 0.60^b^	1.01 ± 0.06	0.73 ± 0.03
*galt5-1*	0.95 ± 0.10^b^	1.17 ± 0.05^b^	0.63 ± 0.05^b^
*galt5-2*	0.90 ± 0.08^b^	1.20 ± 0.07^c^	0.60 ± 0.04^b^
*galt6-1*	1.25 ± 0.09^c^	1.30 ± 0.07^c^	0.55 ± 0.05^b^
*galt6-2*	1.31 ± 0.05^c^	1.29 ± 0.04^c^	0.61 ± 0.08^b^
*galt2galt5*	1.40 ± 0.09^d^	1.36 ± 0.08^d^	0.47 ± 0.05^d^

^a^Intact seeds were extracted sequentially with 0.2 % ammonium oxalate, 0.2 N NaOH and 2 N NaOH, neutralized, and assayed by the phenol-sulfuric acid method against glucose standards. The results are shown as μg/mg of seeds. Analyses were performed in triplicate and results are given as μg/mg seed ± SE. All genotypes were grown, harvested, and stored together. Letters ‘b’ ‘c’ and ‘d’ denote a significantly difference from the wild type (Dunnett’s test, *P* <0.05; *P* <0.01; *P* <0.001 respectively)

### *GALT6* mutants demonstrate premature senescence

Only the *GALT6* mutants (*galt6-1* and *galt6-2*) displayed early onset of senescence compared to WT and the other *galt* mutants. This was visualized by premature yellowing of leaves and was correlated with a slightly greater reduction in chlorophyll content and protein content in *GALT6* mutants compared to WT (Additional file 1: Figure S9). These observations were consistent with the abundance of *GALT6* transcripts in senescent leaves as well as with the markedly greater reduction of β-Gal-Yariv precipitable AGPs in *galt6* senescent leaves (Additional file 1: Figure S4; Table 2).

### *GALT3*, *GALT4,* and *GALT6* mutants exhibit pollen tube and root growth which is less sensitive to β-Gal-Yariv reagent

**Fig. 9:**
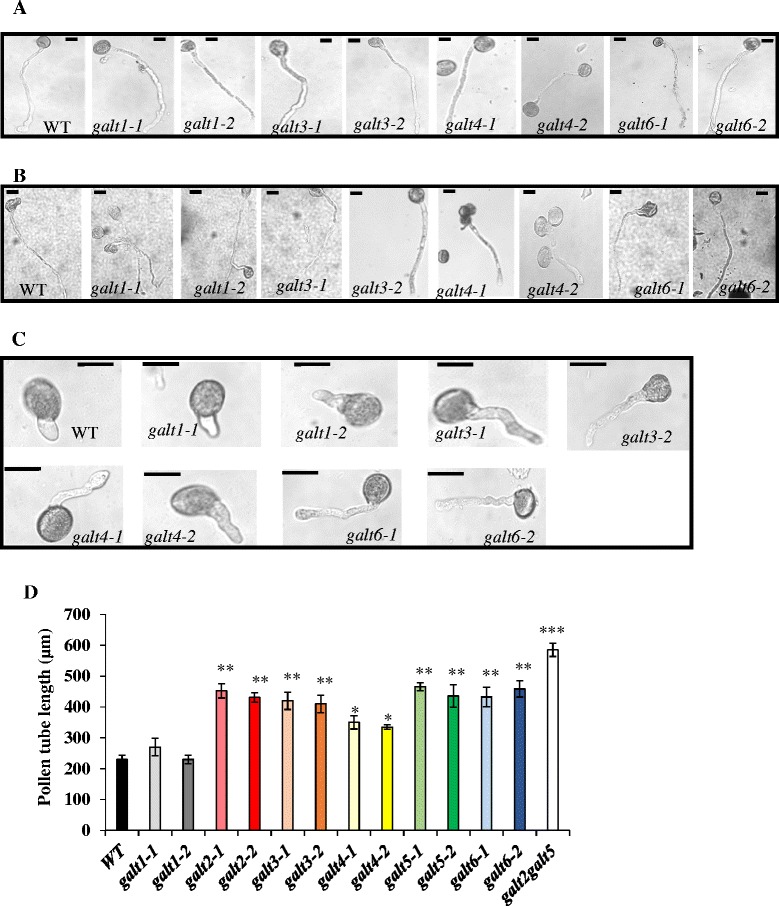
The *galt* single mutants demonstrate reduced inhibition of pollen tube growth in the presence of β-Gal-Yariv reagent. **a** Representative images of pollen tubes from WT, *galt1*, *galt3, galt4*, and *galt6* mutants after 16 h in pollen germination medium, and **b** in pollen germination medium supplemented with 30 μM α-Gal-Yariv, and **c** in pollen germination medium supplemented with 30 μM β-Gal-Yariv reagent. Bar = 50 μm. **d** Pollen tube lengths from WT, *galt1-galt6* mutants, and *galt2galt5* double mutants were measured over 16 h in the pollen germination medium supplemented with 30 μM β-Gal-Yariv reagent. Twenty flowers from each genotype and 25 pollen tubes from each flower were measured using Image J software. The experiment was done in triplicate, and the asterisks indicate mean values significantly different from the WT (Dunnett’s test, *P <0.05; **P <0.01; ***P <0.001)

**Fig. 10:**
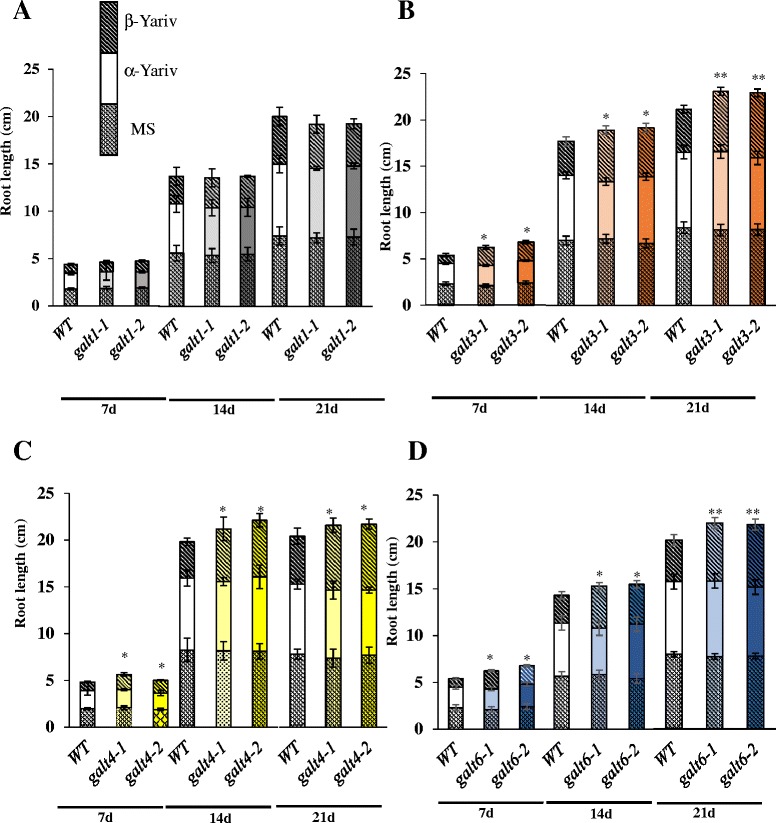
Reduced inhibition of primary root growth of *galt3*, *galt4,* and *galt6* mutants in the presence of β-Gal-Yariv reagent. Root lengths of WT, *galt1*, *galt3, galt4*, and *galt6* plants were measured 7, 14, and 21 days after germination and seedling establishment for 5 days on MS plates, on MS plates supplemented with 50 μM α-Gal-Yariv reagent, and on MS plates supplemented with 50 μM β-Gal-Yariv reagent. Statistical differences were determined by ANOVA, followed by the Tukey’s honestly significant difference test. Asterisks indicate mean values significantly different from the WT expression of the indicated genes within a treatment group (Dunnett’s test, *P <0.05; **P <0.01). Vertical bars represent mean ± SE of the experimental means from at least three independent experiments, where experimental means were obtained from 10 to 15 seedlings per experiment

The *galt3, galt4,* and *galt6* mutants displayed reduced inhibition of pollen tube and root growth elongation in response to β-Gal-Yariv reagent compared to WT or α-Gal-Yariv reagent control treatments (Figs. 9, 10, Additional file 1: Figure S10). As expected, *GALT1* mutants did not exhibit any difference in either pollen tube or root growth elongation compared to WT. Moreover, no significant difference in pollen tube or primary root growth elongation was observed in unsupplemented germination media, indicating the conditional nature of this phenotype (Figs. 9 and 10).

### Conditional salt hypersensitive phenotypes of the *galt* mutants

**Fig. 11:**
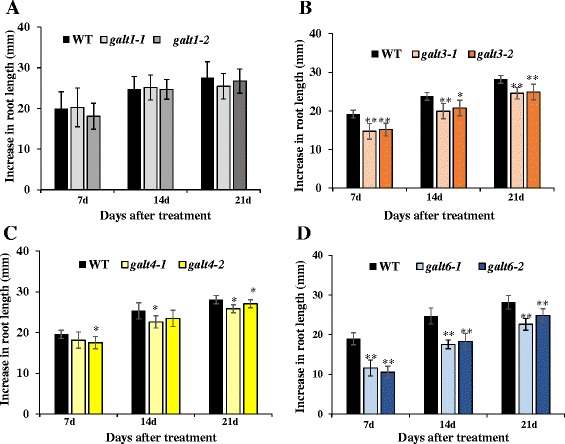
Salt-induced inhibition of primary root elongation in *galt3*, *galt4,* and *galt6* mutants. Five-day-old WT, *galt1*, *galt3, galt4,* and *galt6* seedlings germinated on MS medium were transferred onto media containing 100 mM NaCl and grown vertically. Root elongation (i.e., increase in length after transfer) was measured after 7, 14, and 21 days of growth in the non-permissive media. Data are the means ± SE of measurements from five independent experiments (total *n* = 100). Asterisks indicate mean values significantly different from the WT (Dunnett’s test, *P <0.05; **P <0.01)

*GALT3* (*galt3-1* and *galt3-2*) and *GALT6* (*galt6-1* and *galt6-2*) mutants, and to a lesser extent the *GALT4* (*galt4-1* and *galt4-2*) mutants, exhibited significant reductions in root elongation compared to WT when grown in the presence of 100 and 150 mM NaCl (Fig. 11 and Additional file 1: Figure S11). Such reductions in root elongation were previously reported for knock-out mutants of *GALT2* (*galt2-1* and *galt2-2*), *GALT5* (*galt5-1* and *galt5-2*), and the *galt2galt5* double mutant [16]. As expected, *GALT1* mutants did not show salt hypersensitive growth and were indistinguishable from WT in this assay. The *galt* single and double mutants were not sensitive to osmotic stress as illustrated by mannitol (Additional file 1: Figure S12).

Microscopic examination of the *GALT3*, *GALT6*, and to a lesser extent the *GALT4* mutants also revealed defective anisotropic root tip growth (i.e., root tip swelling) in the presence of 100 mM NaCl, which was not observed in WT and *GALT1* mutants (Additional file 1: Figure S13). Such salt hypersensitive root tip swelling responses were previously reported in *galt2*, *galt5*, and *galt2galt5* mutants and were included here as positive controls [16].

A root bending assay was used as another means to evaluate salt hypersensitivity of the *GALT* mutants (Additional file 1: Figure S14). This assay is commonly used by plant researchers to evaluate salt sensitivity/tolerance and involves monitoring root growth reorientation after a 180 degree reorientation of the seedling to gravity. Results of this experiment indicated that *GALT3* (*galt3-1* and *galt3-2*) and *GALT6* (*galt6-1* and *galt6-2*) mutants, and to a lesser extent the *GALT4* (*galt4-1* and *galt4-2*) mutants were slow to reorient their root growth compared to WT when grown in the presence of 100 mM NaCl. Such delayed reorientation was previously reported for knock-out mutants of *GALT2* (*galt2-1* and *galt2-2*), *GALT5* (*galt5-1* and *galt5-2*), and the *galt2galt5* double mutant; these mutants were used here as positive controls [16]. As expected, *GALT1* mutants (*galt1-1* and *galt1-2*) reoriented quickly and were indistinguishable from WT in this assay.

## Discussion

### *GALT2-GALT6* encode Hyp-GALTs for AGPs and are widely expressed in Arabidopsis

Bioinformatic approaches were previously used to identify a small, six-membered gene family within the GT 31 family of the CAZY database as potential candidates for encoding Hyp-*O*-GALTs for AGPs [15, 18, 42]. Protein members of this family were designated GALT1-GALT6 and distinguished by the presence of a GALT domain as well as a GALECTIN domain. Previously, GALT1 was shown to catalyze galactose addition for formation of the Lewis a epitope on *N*–linked glycans [24], while GALT2 and GALT5 were shown to act as Hyp-GALTs specific for AGPs [15, 16]. In this study, biochemical and genetic evidence indicates that GALT3, GALT4, and GALT6 also act as Hyp-GALTs for AGPs.

Heterologous transient expression of *GALT1-GALT6* in tobacco epidermal cells demonstrated a significant increase in Hyp-GALT activity compared with various tobacco control plants, with the notable exception of *GALT1* (Fig. 1 and Additional file 1: Table S4). The absence of Hyp-*O*-GALT activity over background levels in case of *GALT1* is consistent with its reported, non-AGP related function and provided a useful control for the studies reported here [24]. Moreover, this transient expression study in tobacco corroborates previous findings that GALT2 and GALT5, which were expressed in *Pichia pastoris*, act as AGP-specific Hyp-GALTs [15, 16]. It should be noted that the amount of activity detected in these two heterologous expression systems varied; the tobacco system showed much higher levels of activity than the *Pichia* system, even when taking into account the higher level of endogenous activity associated with the tobacco system. This observation could be explained by the need for other plant-based proteins or factors to enhance or optimize Hyp-*O*-GALT activity.

Substrate specificity of GALT2-GALT6 was investigated using various potential acceptor substrates and demonstrated that GALT2-GALT6 is specific for model AGP sequences (Fig. 2). These findings are consistent with the Hyp contiguity hypothesis, which states that clustered, non-contiguous Hyp residues are sites of AG addition, whereas contiguous Hyp residues are sites for the addition of Ara oligosaccharides [43, 44]. Despite the higher enzyme activity observed in the transient tobacco expression system compared to *Pichia*, the same size-dependent preference for AGP substrates was observed for GALT2 and GALT5 in both systems, where [AO]_7_ was the preferred substrate [16]. GALT3, GALT4, and GALT6 also acted in a similar manner within the tobacco system.

Heterologous transiently expressed *GALT2-GALT6* in tobacco microsomes have similar biochemical properties to the GALT(s) present in Arabidopsis microsomal membranes and to *GALT2* and *GALT5* expressed in *Pichia*, specifically all require UDP-Gal as the sugar donor [15, 16, 45, 46]. They have a requirement for Mn^2+^ followed by Mg^2+^ for their optimal activity, in contrast to Mg^2+^ followed by Mn^2+^ in *Pichia* microsomes.

Genetic mutant analysis provides additional in vivo evidence that GALT3, GALT4, and GALT6 function as Hyp-GALTs, similar to GALT2 and GALT5 (Tables 1 and 2). Allelic *galt* knock-out mutants for all these genes exhibit reduced (i.e., 15–35 % less) Hyp-GALT activity and contain considerably less (i.e., 10–60 % less) glycosylated (i.e., β-Gal-Yariv precipitiable) AGPs. In addition, AGP profiling of the *galt3*, *galt4*, and *galt6* mutants extends these findings and indicates that their activity is not limited to a particular AGP or a small subset of AGPs, but instead broadly acts on coexpressed AGPs, similar to that previously reported for *galt2* and *galt5* mutants (Additional file 1: Figure S7).

qRT-PCR analysis of *GALT1-GALT6* was performed to examine their expression patterns and provide information relevant to phenotypic analysis of their corresponding allelic mutants (Fig. 3). All six genes were widely expressed, and in the cases of *GALT2-GALT6* are consistent with the widespread distribution of AGPs and the multiple functions associated with them. These patterns were corroborated by searching public expression databases, which revealed even broader organ and tissue expression patterns (Additional file 1: Figure S4). HPGT1-HPGT3, three recently identified Hyp-*O*-GALTs for AGPs that lack GALECTIN domains, were also included in this analysis and showed equally broad patterns of gene expression [17]. Nonetheless, within a given organ or tissue, the *Hyp-O-GALT* genes exhibit both temporal and spatial differences in their expression patterns. Transcriptome analyses using RNA extracted from laser-capture dissected seed coat tissue [36] provides a particularly striking illustration of the diverse tissue-specific expression patterns of *GALT2-GALT6* and *HPGT1-HPGT3* (Additional file 1: Figure S5).

### GALT3, GALT4, and GALT6 are localized to Golgi vesicles

Various approaches have indicated that AGP glycosylation occurs in Golgi vesicles. These approaches include bioinformatics predictions using Signal P and Golgi Predictor, biochemical experiments on hydroxyproline-rich glycoprotein (HRGP) biosynthesis [25, 45–49], a proteomics technique for localization of organelle proteins by isotope tagging [49], and localization studies performed with other AGP GTs, including GALT2, GALT5, HPGT1-HPGT3, AT1G77810, GALT31A, GALT29A, GlcAT14A, and FUT6 [12, 15, 16, 18, 19, 21]. Given their similarity to GALT2 and GALT5 and their demonstrated Hyp-GALT activity, GALT3, GALT4, and GALT6 were expected to reside in the Golgi vesicles, and this was confirmed by heterologous expression of fluorescently tagged protein fusions in tobacco leaves (Fig. 4). Interestingly, only GALT2 is found in both the ER and Golgi, indicating that Hyp-galactosylation may be initiated in the ER, but completed in the Golgi where the bulk of the Hyp-*O*-GALTs are located [15].

### GALT mutant phenotypes reveal functional roles of AGP glycosylation in normal growth and development

Genetic mutant analysis was used to investigate and compare the in vivo functional contributions of AGP glycosylation by *GALT3*, *GALT4,* and *GALT6* with that of *GALT2* and *GALT5* (Fig. 5; Additional file 1: Table S1). To date, a variety of functions are attributed to certain AGPs or GTs acting on AGPs based on mutant analysis; these mutants show embryo lethality, conditional defects of primary root growth, cell elongation, and pollen tube growth (Additional file 1: Table S5) [13, 14, 16, 17, 19, 21, 23]. Like *galt2* and *galt5, galt3*, *galt4*, and *galt6* single mutant lines showed subtle or no detectable growth phenotypes under normal soil-based growth conditions, which is likely attributed to the functional redundancy within the *GALT2-GALT6* gene family [15, 16]. The phenotypes that were displayed by the single mutants here included reduced root hair length and/or density for *galt3*, *galt4*, and *galt6* (Fig. 6), reduced seed set for *galt4* and *galt6* (Fig. 7; Table 3), reduced adherent seed mucilage for *galt3,* and *galt6* (Fig. 8 and Table 4), and accelerated leaf senescence for *galt6* (Additional file 1: Figure S9). Although these phenotypes are consistent with the expression profiles of these genes, it would be difficult to predict such phenotypes from expression data alone. It is anticipated that double and multiple *galt* mutants will show more pronounced mutant phenotypes, as was the case when *galt2galt5* double mutants were produced and characterized [16].

With respect to root hair length and density, knock-out mutants of *GALT3* and to a lesser extent GALT6 and GALT4, displayed shorter and/or less dense root hairs, indicating glycosylated AGPs play a role in tip growth of root hairs in *Arabidopsis* (Fig. 6). The *galt2*, *galt5*, and *galt2galt5* double mutants also demonstrate this response and were used here as positive controls [16]. Reduced density of root hairs can be attributed to an increase in longitudinal length of epidermal cells and/or to a decrease in the number of root hair-forming cells leading to the formation of less root hairs [50, 51]. Other studies, using β-Gal-Yariv reagent and prolyl hydroxylase genetic mutants, have also indicated that HRGPs (i.e., AGPs and extensins) are involved with root hair growth [52–55].

With respect to seed mucilage, *galt3* and *galt6* mutants display the most significant decrease in the width of the adherent seed mucilage layer, along with *galt2* and *galt5* upon staining with ruthenium red when compared to WT seeds, which indicates that glycosylated AGPs are involved in maintaining the adherent mucilage layer in seeds (Fig. 8, Tables 4 and 5). The *galt2galt5* double mutants also demonstrate this response and were used here as positive controls [16]. Harpaz-Saad et al. [37] reported that both *FEI2*, a cell wall leucine–rich receptor-like kinase and SOS5, a fasciclin like AGP are critical for the synthesis and proper deposition of cellulosic rays in seed coat mucilage, which coincides with an increase in solubility of the pectinaceous component of seed coat mucilage. The *GALT6* expression profile during seed coat differentiation is identical to that of *FEI2*. Moreover, *GALT6* is expressed at a higher level during late embryogenesis compared to the early embryogenesis stages, consistent with its involvement with seed coat development and in seed coat mucilage adherence. Taken together, GALT6, GALT3, GALT2 and GALT5 likely glycosylate AGPs, like SOS5, that are essential for maintaining mucilage adherence in seeds during hydration.

With respect to seed set, *galt4* and *galt6* mutants phenocopied knock-out mutants of two pollen-specific AGPs genes, *AGP6* and *AGP11*, in terms of reduction in number of seeds per silique, but not in abnormal pollen structures, although occasionally some collapsed pollen were observed in *galt4* and *galt6* mutants (Fig. 7, Table 3, Additional file 1: Figure S8) This suggests GALT4 and GALT6 may be involved in glycosylation of pollen -specific AGPs. Several studies have indicated that AGPs play key roles in pollen biogenesis, pollen tube growth and development, pollen tube guidance, and pollen–pistil interactions during post pollination events [56–61]. Indeed, a number of AGP genes are reported to show pollen-specific expression in Arabidopsis, including *AGP6*, *AGP11*, *AGP23,* and *AGP40*. Reciprocal crosses of the mutants with WT confirmed that genetic transmission of this phenotype is contributed by the male gametophyte (Fig. 7 and Table 3). In contrast, it is interesting to note that *AGP18* is reported to be essential for female gametogenesis, given that functional megaspores in RNAi plants fail to enlarge and divide, resulting in ovule abortion and reduced seed set [62].

With respect to leaf senescence, *galt6* mutants displayed age-dependent early onset of leaf senescence, indicating AGP glycosylation is related to plant aging (Additional file 1: Figure S8 and Table 2). Several lines of evidence implicate the involvement of AGPs in regulating programmed cell death and senescence [63–67]. For example, β-Gal-Yariv treatment is known to promote programmed plant cell death, while overexpressing AGPs results in tomato plants with enhanced lifespans [67]. In this context, it is noteworthy that GALT6 is highly expressed in senescing leaf tissue and that only *galt6* mutants demonstrate a significant reduction in the β-Gal-Yariv precipitable AGPs in senescent leaves (Additional file 1: Figure S4 and Table 2).

Any one defect leading to reduced AG glycosylation is likely to impair the function of multiple AGPs, leading to pleiotropic effects as observed here. At least some of the genes involved in AGP glycosylation, however, exist in small redundant or partially redundant genes families and may compensate for one another when a given gene in the family is knocked out [12, 15–17, 21, 22]. In some cases, aberrant phenotypes may not be discernable under normal conditions, but may be revealed under suboptimal growth conditions.

### Conditional phenotypes indicate *GALT3, GALT4,* and *GALT6* function in tip growth

The *galt3*, *galt4*, and *galt6* mutants display several conditional pollen and root phenotypes in response to β-Gal-Yariv treatment or salt treatment similar to those observed in *galt2* and *galt5* single mutants as well as *galt2galt5* double mutants [16]. In both pollen tubes and roots, β-Gal-Yariv treatment is known to bind AGPs, specifically to their β-1,3-galactan chains, and inhibit pollen tube and root elongation [68–71] indicating AGPs are important for such growth. This inhibition is alleviated in single mutants, which have reduced AGP glycosylation due to the lack of respective GALTs (Figs. 9, 10, Additional file 1: Figure S10 and Figure S11). Under normal conditions (i.e., without β-Yariv treatment), no inhibition is observed in the single mutants, most likely due to gene redundancy, a conclusion supported by the observation that *galt2galt5* double mutants show inhibition under normal conditions [16]. Thus, GALT3, GALT4, and GALT6, like GALT2 and GALT5, are important for pollen tube and root growth and indicate that the AG polysaccharides are required for these growth functions.

In roots, salt treatment results in reduced root growth, which can be measured directly or by the root bending assay, which is commonly used to screen plants or mutants for salt sensitivity. Here, *galt3*, *galt4*, and *galt6* mutants display salt hypersensitive root growth in both of these assays (Fig. 11). Thus, GALT3, GALT4, and GALT6, like GALT2 and GALT5, function in root growth and indicate the importance of AG polysaccharides in this process. GALT3, GALT4, and GALT6, like GALT2 and GALT5, also function to prevent root tip swelling in response to salt stress (Additional file 1: Figure S13 and Figure S14). Since SOS5, a fasciclin-like AGP, and FEI1 and FEI2, two cell wall receptor-like kinases, also prevent such root tip swelling, and are in the same genetic pathway as GALT2 and GALT5, it appears that AGP glycosylation via any of the five GALTs (GALT2-GALT5) likely generates a carbohydrate signal on SOS5, which is detected and transduced by the FEI1/FEI2 kinases to promote cell wall integrity [16, 72, 73].

## Conclusions

In conclusion, biochemical and genetic evidence presented here indicates that GALT3, GALT4, and GALT6, like GALT2 and GALT5, function as AGP-specific Hyp-GALTs. The largely, but not completely, overlapping pleiotropic effects observed in genetic null mutants for each of the genes with respect to multiple aspects of plant growth, development, and reproduction indicate the importance of AG polysaccharides to the functions of AGPs. Thus, these *Hyp-GALT* genes function in a largely redundant manner, and it is anticipated that more severe biochemical and physiological phenotypes will occur when multiple genetic mutants are studied, revealing additional AGP functions. Indeed, future work aimed at examining such multiple mutants, the role of the GALECTIN domain, the potential for enzyme complex formation between and among Hyp-GALTs and other GTs involved with AGP biosynthesis, Hyp-GALT/AGP trafficking, and the potential signaling roles of AG polysaccharides will provide deeper insight to the evolution and biology of this small enzyme family and the AGP family members that serve as their substrates.

## Methods

### *In silico* analysis of the six-membered GALT family

Protein sequences from GALT1, GALT3, GALT4 and GALT6 were run through several prediction programs (TMHMM 2.0, TargetP 1.1, SignalP v2.0.b2 server) to obtain information on their putative subcellular localization and topology [25, 26, 74]. Hydrophobic cluster analysis (HCA) plots were obtained from the drawhca server on the Internet (http://bioserv.impmc.jussieu.fr/hca-seq.html) and were analyzed as described by Breton et al. [75]. The coexpression network for the GT31 member genes was illustrated using the program GENEMANIA (www.genemania.org) using *GALT2* and *GALT5* as query genes.

### Plant lines and plant growth conditions

Arabidopsis thaliana accession Columbia-0 (Col-0) and two T-DNA insertion lines for *At1g26180*-(*galt1-1*, Sail_170_A08 and *galt1-2,* Salk_006871), *At3g06440* (*galt3-1*, Salk_085633 and *galt3-2,* Salk_005178), *At1g127120 (galt4-1*, Salk_136251 and *galt4-2*, Salk_131723), and *At5g62620 galt6-1,* Sail_59_D08 and *galt6-2,* Sail_70_B02) were obtained from the Arabidopsis Biological Resource Center (ABRC, Ohio State University). All plants used in this study were germinated after 4 days of stratification in the dark at 4 °C and were grown under long-day conditions (16 h of light/8 h of dark, 22 °C, 60 % humidity) in growth chambers or growth rooms. Sequencing of the amplified PCR products from the mutant plants led to the accurate determination of the T-DNA insertion site.

### Mutant confirmation by PCR and RT-PCR

Genomic DNA was isolated from leaves of the mutants and WT plants and was extracted using the 2× CTAB method described by Murray and Thompson [76]. Subsequent PCR analysis was carried out using gene specific primers in conjunction with the T-DNA primers. The primer locations are indicated in Fig. 4, and the corresponding primer sequences are listed in Additional file 1: Table S6. For sequencing, PCR products were purified by gel extraction (Wizard® SV Gel and PCR Clean-Up System, Promega, Madison, WI, USA) and sequenced by the Ohio University Genomics Facility (http://www.dna.ohio.edu/). To analyze transcript levels of *GALT1*, *GALT3*, *GALT4*, and *GALT6*, total RNA was isolated from 2 week old seedlings of WT and mutant plants. For tissue specific expression profiling, tissue was harvested at different growth stages as defined by Boyes et al. [77]. In both cases, RNA was extracted using Trizol (Life Technologies, Grand Island, NY, USA) and Direct-zol™ RNA MiniPrep kit (Zymo Research, Irvine, CA, USA). First-strand cDNA synthesis was performed from 2 μg of total RNA using oligo-dT (Coralville, Iowa) and GoScript reverse transcriptase (Promega, Madison, WI, USA). RT-PCR was performed using OneTaq DNA polymerase (New England Biolabs, Ipswich, MA, USA) and gene-specific RT primers (Additional file 1: Table S6). The number of amplification cycles was 28 to evaluate and quantify differences among transcript levels before the reaction reached saturation.

For quantitative real-time PCR (qRT-PCR), cDNAs were amplified using Brilliant II SYBR Green qRT-PCR Master Mix with ROX (Agilent Technologies, La Jolla, CA, USA) in an MX3000P real-time PCR instrument (Agilent Technologies). PCR was optimized and reactions were performed in triplicate. The transcript level was standardized based on cDNA amplification of ubiquitin 10 (*UBQ10*, *At4g05320*) as a reference. Primer sequences are listed in Additional file 1: Table S6.

### Heterologous expression of *GALT1*, *GALT3*, *GALT4*, and *GALT6* and Hyp-GALT activity assay

Coding regions of *GALT1, GALT3,* and *GALT4* were obtained from the RIKEN Bioresource Center, while the coding region of *GALT6* was obtained from The French Plant Genomic Resource Center (http://cnrgv.toulouse.inra.fr/). N-terminal 6x-His tag fusion gene constructs were amplified using Q5 high fidelity DNA Taq polymerase (New England Biolabs, Ipswich, MA, USA), initially cloned into the pENTR/D-TOPO vector (Life technologies, Grand Island, NY, USA), and eventually cloned into the destination vector pMDC32 gateway vector using LR clonase enzyme mix (Life Technologies Grand Island, NY, USA). Primers used for amplification are listed in Additional file 1: Table S6. Gene constructions were transformed into *Agrobacterium* strain GV3101 by the freeze thaw method, and the transformants were grown overnight in Luria-Bertani (LB) medium. Bacterial cells were harvested by centrifugation and suspended in a buffer containing 10 mM MES, 10 mM MgCl_2_, and 50 μM acetosyringone (OD_600_ = 0.2). Leaves from 6-week-old WT *N. tabacum* cv. Petit Havana were used for *Agrobacterium*-mediated transient expression. Four days after infiltration, leaves were harvested, and microsomes were prepared according to the method described by Liang et al. [45] with minor modifications.

### Fluorescent protein fusion and subcellular localization

Full length *GALT3*, *GALT4*, and *GALT6* devoid of stop codons was cloned into the pENTR/D-TOPO vector (Life technologies, Grand Island, NY, USA) and sequenced. The resulting plasmids were cloned in the destination vector pEarlyGate 101 by a gateway cloning strategy, using LR clonase enzyme mix (Life Technologies Grand Island, NY, USA) to generate the YFP N-terminal fusion constructs. These gene constructions were transformed into *Agrobacterium* strain GV3101 and infiltrated into tobacco leaves as described in the above section except that the bacterial concentration was lower (OD_600_ = 0.05). The *GALT3-YFP*, *GALT4-YFP*, and *GALT6-YFP* constructions were co-expressed with either the ER marker HDEL-GFP or the Golgi marker sialic acid transferase (ST)-GFP to ascribe subcellular localization. Transformed plants were incubated under normal growth conditions and imaged 2 days post-infiltration using an upright Zeiss LSM 510 META laser scanning microscope (Jena, Germany), with a 40 × oil immersion lens and an argon laser. For imaging the expression of YFP constructs, the excitation line was 514 nm, and emission data were collected at 535–590 nm; whereas for GFP constructs, the excitation line was 458 nm, and the emission data were collected at 505–530 nm.

### GALT assay with microsomal preparations from transiently expressed *GALT1, GALT3, GALT4,* and *GALT6* in tobacco epidermal cells

The standard GALT reaction was performed as described in Basu et al. [15] using detergent permealized microsomes from transiently expressed *GALT1*, *GALT3*, *GALT4*, and *GALT6*. Three permeabilized microsomal membranes were included as controls, one from the WT tobacco leaves, one from WT tobacco leaves infiltrated with *Agrobacterium* GV3101 transformed with the empty expression vector (pMDC32), and one with tobacco leaves infiltrated with ST-GFP constructs as negative controls.

### Purification of Hyp-GALT reaction products by reverse-phase HPLC

The GALT reaction products were purified by RP-HPLC as described by Liang et al. [45].

### Determination of substrate specificity for GALT2-GALT6

Microsomal fractions from tobacco leaves expressing *GALT2-GALT6* constructs were used for determination of substrate specificity as described by [15, 16].

### Isolation of Golgi-enriched plant microsomal membranes

Plant microsomal membranes were extracted from WT, *galt1-1*, *galt1-2*, *galt3-1*, *galt3-2*, *galt4-1*, *galt4-2*, *galt6-1* and *galt6-2* according to Liang et al. [45] with minor modifications.

### Extraction of AGPs

AGPs were extracted from the WT, *galt1-1*, *galt1-2*, *galt3-1*, *galt3-2, galt4-1, galt4-2, galt6-1,* and *galt6-2* mutant plants as described by Schultz et al. [78] precipitated and quantified as described by Gao et al. [79] and Yariv et al. [80]. AGP profiling was conducted as described by Youl et al. [81] with modifications. AGPs were obtained from 8 g of plant material, precipitated by β-Gal - Yariv reagent and dissolved in 1 ml of deionized water before applying 100 μl onto a polymeric reverse-phase column (PRP-1, 5 μm, 4.1 × 150 mm; Hamilton) equilibrated with buffer A (0.1 % trifluoroacetic acid). Fifty μg of [AO]_7_ was used as a control to monitor the retention time of a pure AGP peptide. Samples were eluted from the column following a linear gradient with solvent B (0.1 % trifluoroacetic acid in 80 % acetonitrile): 0–30 % solvent B in 30 min, then 30–100 % in 30 min at a flow rate of 0.5 ml/min. Chromatography was monitored by absorption at 215 and 280 nm.

AGPs from siliques, flowers, inflorescence stem, and senescent leaves were extracted as described by Lamport [82], with minor modifications. To extract AGPs from siliques (2.5 g), flowers (1.5 g), inflorescence stems (5 g), and senescent leaves (5 g), they were ground to a fine powder in liquid nitrogen. Ground tissue was added to an extraction buffer of CaCl_2_ (2 % w/v) at a volume of 2 ml for each gram of tissue, and stirred for 3 h at room temperature. Samples were centrifuged for 30 min at 10,000 g at room temperature. The supernatant was freeze-dried overnight and resuspended in 1 ml of 2 % CaCl_2_ and transferred to 2 ml microcentrifuge tubes. AGPs were precipitated overnight at 4 °C with an equal volume of the β-Gal - Yariv reagent (2 mg/m in 2 % w/v CaCl_2_). The insoluble β-Gal-Yariv–AGP complex was collected by centrifugation at 10,000 g in a microcentrifuge for 1 h. The β-Gal-Yariv was removed by washing the pellet three times in 2 % (w/v) CaCl_2_ and then twice in methanol. The pellet was dried, dissolved in 100 μl of water mixed with 25 mg of solid sodium dithionite and incubated for 30 min at 50 °C until the mixture decolorized. The resulting solution was then desalted on a PD-10 column (Pharmacia) that had been equilibrated with water, and the eluate was freeze-dried.

### Evaluation of seed set

Mature siliques from 6-week old WT, *galt1*, *galt3*, *galt4,* and *galt6* plants were collected, and silique length and weight were measured. For seed number, siliques were decolorized by incubation in 100 % ethanol at 37 °C overnight before dissection of the siliques. For reciprocal cross-pollinations, 10 flowers from WT, *galt4-1*, *galt4*-2, *galt6-1,* and *galt6-2* were selected at stage 12. These flowers were emasculated before pollinating them with fresh pollen obtained from flowers at stage 13. After 10 days, siliques were collected from these flowers to examine seed set.

### Root growth measurements

For monitoring root growth in response to β-Gal-Yariv reagent, WT, *galt1-1*, *galt1-2*, *galt3-1*, *galt3-2*, *galt4-1*, *galt4-2, galt6-1* and *galt6-2* seedlings were grown on MS plates for 7 days before they were transferred to MS plates supplemented with 50 μM α-Gal-Yariv reagent or 50 μM β-Gal-Yariv reagent. For seedling growth in salt, 7-day-old seedlings of WT, *galt1-1*, *galt1-2*, *galt3-1*, *galt3-2, galt4-1, galt4-2, galt6-1,* and *galt6-2* were transferred to MS medium containing 1 % agar and 100 mM or 150 mM NaCl. Root length was determined on low-magnification (×10) digital images captured using a CCD camera and image analysis freeware (ImageJ; http://rsb.info.nih.gov/ij/). For analysis of salt hypersensitivity of the mutant plants, root growth was monitored using a root bending assay [83], and images were taken using a Nikon SMZ1500 stereomicroscope coupled with a CCD Infinity 2 camera and analysis software.

### In vitro pollen germination assay

Flowers collected from WT, *galt1-1, galt1-2*, *galt3-1*, *galt3-2, galt4-1, galt4-2, galt6-1*, and *galt6-2* plants 1 to 2 weeks after bolting were used for the examination of pollen tube phenotypes. Individual open flowers were germinated in vitro as described by Boavida and McCormick [84], on solid germination medium (0.01 % H_3_BO_3_, 1 mM MgSO_4_, 5 mM KCl, 5 mM CaCl_2_, 10 % sucrose, and 1.5 % low-melting agarose, pH 7.5 and 30 μM β-Gal-Yariv reagent or 30 μM α-Gal-Yariv reagent) at 22 °C and 100 % humidity in the dark. Pollen tube germination rates were calculated by dividing the total number of germinated tubes by the number of grains. Images and measurements of pollen tubes were done at 20× magnification in a Nikon Phot-lab2 microscope coupled with a SPOT RT color CCD camera and SPOT 4.2 analysis software.

### Aberrant root hair morphology

Root hair length from 8-day-old plants grown on agar plates was determined on low-magnification (×10) digital images captured using a CCD camera and image analysis freeware (ImageJ; http://rsb.info.nih.gov/ij/). To ensure comparable results, the area 3–5 mm behind the root tip was analyzed. Plants grown on agar plates were carefully removed in 100 μl of half-strength MS medium on microscope slides for analysis. Quantification of root hairs length and density was performed using 10 seedlings for each genotype, and 25 root hairs from each root were measured.

### Cytochemical staining of seeds and determination of adherent mucilage size and mass

Seeds of all the indicated genotypes were prehydrated in water and stained with 0.01 % ruthenium red. The staining was performed as described by Willats and Knox, [85] and Harpaz-Saad et al. [37]. Imaging was done using a Zeiss LSM 510 confocal microscope. The volume of adherent mucilage was measured using the method described by Yu et al. [86].

### Alexander’s staining of pollen

To examine pollen viability, anthers were removed from flowers, and mounted on microscope slides, and stained with Alexander’s stain as described by Alexander [87].

### Leaf senescence assay

Leaf 7 was marked as 18 DAG (days after germination) and sampling started at 19 DAG and continued every other day until full senescence was reached (39 DAG) as described by Breeze et al. [88]. Because the age of a leaf can affect its response to factors that influence senescence, plants of indicated genotypes were taken from synchronously growing populations, and only identically aged leaves were pooled for chlorophyll and protein content measurements.

### Measurement of chlorophyll content

Chlorophyll was extracted from WT, *galt6-1,* and *galt6-2* leaves by immersion in 1 ml of N, N-dimethylformamide for 48 h in the dark at 4 °C. Absorbance was recorded at 664 and 647 nm, and total chlorophyll concentration was calculated as described by Xiao et al. [89]. The total chlorophyll content was measured and normalized per gram fresh weight of sample.

### Cell wall preparation

One-hundred milligram of WT and *galt* mutant seeds were extracted sequentially with 0.2 % ammonium oxalate, 0.2 and 2 N sodium hydroxide for 1 h each with vigorous shaking at 37 °C. Both sodium hydroxide extractions were neutralized with acetic acid. Total sugar (μg/mg seed) was determined with a phenol-sulfuric assay based on Dubois et al. [90]. In short, 200 μl of resuspended extract was incubated with 100 μl freshly made 5 % (v/v) aqueous phenol and 1 ml concentrated sulfuric acid for 2 h at 30 °C. Absorbance was detected at 500 nm against glucose standards of 0.5, 2.5, 5, 7.5, 10, 15, 25 μg for which a linear response curve was obtained.

### Statistical analysis

For each analysis, both enzyme activity and phenotypic differences in mutants were compared with the WT using one-way ANOVA. The P values were derived from post hoc tests using Dunnett’s adjustment for multiple comparisons. Statistical analyses were performed with Prism 6 software (GraphPad Software, Inc.).

## Availability of supporting data

The data sets supporting the results of this article are included within the article and its additional files. The accession numbers of the genes analyzed in this study are as follows: *GALT1: At1g26810; GALT2: At4g21060; GALT3: At3g06440; GALT4: At1g27120; GALT5: At1g74800; GALT6: At5g62620.*

## Additional file

Additional file 1:
**Six supplemental tables, 14 supplemental figures and their corresponding legends. Table S1.** *GALT1*, *GALT3*, *GALT4*, and *GALT6* mutant and coding region information. **Table S2.** Amino acid identity/similarity among the predicted amino acid sequences of GALT1-GALT6 and HPGT1-HPGT3 to GT31 family by MATCHER (http://mobyle.pasteur.fr/). **Table S3.** List of candidate AGP specific glycosyltranferases and Arabidopsis AGPs coexpressed with *GALT2* and *GALT5* as query genes using the Gene CAT coexpression tool. **Table S4.** Subcellular distribution of Hyp-GALT activity obtained from GALT1-GALT6 transiently expressed in N. tabacum. **Table S5.** List of AGP-specific glycosyltransferases and AGPs with their respective mutant phenotypes [15–22, 37, 57, 60, 62, 69, 91–97]. **Table S6.** List of primers used in this study. **Figure S1.** Predicted transmembrane regions of GALT1-GALT6 and HPGT1-HPGT3. **Figure S2.** Hydrophobic cluster (HCA) analysis of GALTs showing the DXD motif within a pocket of hydrophobic amino acids. **Figure S3.** Biochemical characterization of Hyp-GALT activity. **Figure S4.** Gene expression profile of *Hyp-GALTs* and *GALT1* in different organs/tissues. **Figure S5.** Transcript levels of *Hyp-GALTs* in the developing seed coat depicted by http://seedgenenetwork.net/ [36]. **Figure S6.** Single infiltration in tobacco epidermal cells. Tobacco leaves were infiltrated with either with ST-GFP, HDELGFP, or GALT2-YFP as indicated. Size bar = 10 μm. **Figure S7.** RP-HPLC profiles of AGPs extracted from WT and single *galt* mutants. **Figure S8.** Pollen viability, pollen germination frequency and pollen tube growth of *galt4* and *galt6* mutants. **Figure S9.** Age-dependent leaf senescence phenotype of *galt6-1* and *galt6-2* mutant plants. **Figure S10.** Representative images of WT and *galt* roots treated with 50 μM β -Gal-Yariv reagent. **Figure S11.** Representative images of WT, *galt1*, *galt3*, *galt4*, and *galt6* plants after 14 days of growth on MS plates supplemented with 100 mM NaCl. **Figure S12.** The *galt* mutants are insensitive to mannitol stress. **Figure S13.** Conditional root anisotropic growth defects of *galt2-galt6* mutants and *galt2galt5* double mutants compared to WT and *galt1* plants. **Figure S14.** Root-bending assay of WT, *galt1*, *galt3*, *galt4*, and *galt6* mutant seedlings. (PDF 2122 kb)

## Abbreviations

AGP: arabinogalactan - protein
CAZy: carbohydrate active enzyme database
GT: glycosyltransferase
HRGP: hydroxyproline-rich glycoprotein
Hyp: hydroxyproline
Hyp-*O*-GALT: hydroxyproline–*O*-galactosyltransferase
