# Supplementary material for: A small multigene hydroxyproline-O-galactosyltransferase family functions in arabinogalactan-protein glycosylation, growth and development in Arabidopsis
Source: BMC Plant Biol. 2015 Dec 21;15:295. doi: 10.1186/s12870-015-0670-7 (PMC4687291; doi:10.1186/s12870-015-0670-7)

Additional Files

Additional File 1. Six supplemental tables, fourteen supplemental figures and their corresponding legends.

Supplemental Table 1. GALT1, GALT3, GALT4, and GALT6 mutant and coding region information.

Supplemental Table 2. Subcellular distribution of Hyp-GALT activity obtained from GALT1-GALT6 transiently expressed in *N. tabacum*.

Supplemental Table 3. List of candidate AGP specific glycosyltranferases and Arabidopsis AGPs coexpressed with *GALT2* and *GALT5* as query genes using the Gene CAT coexpression tool.

Supplemental Table 4. Subcellular distribution of Hyp-GALT activity obtained from GALT1-GALT6 transiently expressed in *N. tabacum*.

Supplemental Table 5. List of AGP-specific glycosyltransferases and AGPs with their respective mutant phenotypes.

Supplemental Table 6. List of primers used in this study.

Supplemental Figures

Supplemental Figure 1. Predicted transmembrane regions of GALT1-GALT6 and HPGT1-HPGT3. Sequence analysis using the TMHMM2.0 program for prediction of transmembrane helices of five confirmed Hyp-*O*-GALT proteins namely GALT2, GALT5, HPGT1, HPGT2, and HPGT3, and three putative Hyp-*O*-GALTs namely, GALT3, GALT4 and GALT6. GALT1 is reported to be involved in the addition galactose for formation of the Lewis a epitope on *N*–linked glycans [24].

Supplemental Figure 2. Hydrophobic cluster (HCA) analysis of GALTs showing the DXD motif within a pocket of hydrophobic amino acids. Protein sequences are represented on a duplicated α-helical net, and the clusters of contiguous hydrophobic residues (V, I, L, F, M, W, and Y) are boxed. Gly, Pro, Ser, and Thr, are represented by symbols: Gly (filled diamond), Pro (red star), Ser (square with a black dot), and Thr (empty square). Vertical lines delineate the hydrophobic pocket in which the DXD motif (highlighted in red elipse) can be found. The six GT31 family members containing both GALT and GALECTIN (i.e., GALT1-GALT6) were used in this analysis along with two previously reported GALTs, At1g77810 [18] and GALT31A [19].

Supplemental Figure 3. Biochemical characterization of Hyp-GALT activity.

(A) Effect of different divalent ions on enzyme activity. Tobacco leaves infiltrated with empty vector were used as WT and buffer with no ions was considered as control. (**B)** Specificity of GALT3, GALT4, and GALT6 enzymes for nucleotide sugar donors was analyzed by monitoring the incorporation of ^{14}C-radiolabeled Gal onto $(AO)_7$ substrate acceptor in the presence of UDP-[^{14}C]Glc, UDP-[^{14}C]Gal, UDP-[^{14}C]Xyl, and GDP-[^{14}C]Fuc. Experiments were performed in duplicate. Error bars represent ± SD.

Supplemental Figure 4. Gene expression profile of Hyp-GALTs and GALT1 in different organs/tissues. (**A)** A Genevestigator developmental expression plot of the indicated genes [30]. (**B)** Transcript profiling of the indicated genes using microarray data of semi-*in vitro* germination of pollen tubes [34]. (**C)** Analysis of developmental expression using the eFP browser [36].

Supplemental Figure 5. Transcript levels of Hyp-GALTs in the developing seed coat depicted by http://seedgenenetwork.net/ [36]. Five stages of seed development were monitored for investigating gene networks in seed, namely pre-globular, globular, heart stage, linear cotyledon and mature green seed stage. PEN-Peripheral endosperm, GSC-General seed coat, EP-Embryo proper, S-Suspensor, ME-Micropylar endosperm, CE-Chalazal endosperm, and CSC-Chalazal seed coat.

Supplemental Figure 6. Single infiltration in tobacco epidermal cells. Tobacco leaves were infiltrated with either with ST-GFP, HDEL-GFP orGALT2-YFP as indicated. Size bar=10μm.

Supplemental Figure 7. RP-HPLC profiles of AGPs extracted from WT and single *galt* mutants. RP-HPLC profiles of AGPs prepared from 14-d-old seedlings of WT and *galt* mutant lines by precipitation with β-Gal-Yariv. Arrows indicate the most prominent AGP peaks in the chromatographs.

Supplemental Figure 8. Pollen viability, pollen germination frequency and pollen tube growth of *galt4* and *galt6* mutants. (A) Pollen viability using Alexander's staining**.** Size Bar = 200 μm. **(B)** Pollen germination frequency of the *galt* mutants compared to WT. The germination rates were calculated as the number of germinating pollen divided by the number of pollen sown multiplied by 100. Error bars indicate ± SE (n = 100). **(C)** Determination of pollen tube length at 4, 8 and 16h in pollen germination media. Error bars indicate ± SE (n = 50). There was no significant difference at $P < 0.05$ by Dunnett's test.

Supplemental Figure 9. Age-dependent leaf senescence phenotype of *galt6-1* and *galt6-2* mutant plants. (A) Progression of leaf senescence. Representative WT, *galt6-1* and *galt6-2* leaf 7 was sampled from each plant and was shown from 20 days after germination (DAG) to 37 DAG at 2-day intervals. The leaves were detached and arranged according to their age. **(B)** and **(C)** depict enlarged images of 32 and 34 d old senescent leaves. **(D)** Total protein content was measured from leaf samples at each stage of development. **(E)** Chlorophyll content was measured from the WT and mutant leaves. Values shown in **(D)** and **(E)** represent the means $\pm$ SD of three independent biological replicates per time point. Asterisks indicate significant difference from the wild type at the same age (Dunnett's test; $*P < 0.05$).

Supplemental Figure 10. Representative images of WT and *galt* roots treated with 50 µM β-Gal-Yariv reagent. (A) *galt1*, **(B)** *galt3,* **(C)** *galt4* and **(D)** *galt6* plants. Seeds of the indicated genotypes were stratified for 3d followed by seedling establishment for 5 d in unsupplemented MS media. The 5-old-seedling were transferred to MS plates supplemented with β-Gal-Yariv reagent and were grown for 7 d. The arrow indicates the root tips. Size bar = 1 cm.

Supplemental Figure 11. Representative images of WT, *galt1*, *galt3*, *galt4* and *galt6* plants after 14 d of growth on MS plates supplemented with 100 mM NaCl. Photographs were taken 5 d after NaCl treatment. **(A)** *galt1*, **(B)** *galt3* **(C)** *galt4* and **(D)** *galt6.* Size bar = 1 cm.

Supplemental Figure 12. The *galt* mutants are insensitive to mannitol stress.

Four-day-old seedlings of WT, *galt1-galt6* single mutants, and the *galt2galt5* double mutants were transferred to MS medium (control) and MS medium supplemented with various concentrations

of mannitol (100, 200, 250 and 300 mM); root length measurements were recorded after 7 days of growth. Error bars indicate standard deviations (n = 25). There was no significant difference at P < 0.05 by Dunnett's test.

Supplemental Figure 13. Conditional root anisotropic growth defects of *galt2-6* mutants and *galt2galt5* double mutants compared to WT and *galt1* plants. (A) Light microscopic images of root tips of plant seedlings from indicated genotypes grown for 7 d in MS plates with 100 mM NaCl. Seeds were germinated in MS plates and grown for 3d before transferring the seedlings to MS plates supplemented with 100 mM NaCl. Bar =1mm. (**B)** Analysis of root tip width in WT and *galt* mutants. Quantitation of root tip width was measured at the level of the youngest root hair using ImageJ software. Statistical differences were determined by one-way ANOVA. Values are the means (n>15) ± SE. Asterisks indicate values significantly different from the WT (Dunnett's test, * P <0.05*; ** P <0.01; *** P <0.001).

Supplemental Figure 14. Root-bending assay of WT, *galt1*, *galt3*, *galt4*, and *galt6* mutant seedlingsFive-day-old seedlings grown on MS plates were transferred to MS plates with 100 mM NaCl and reoriented at an angle of 180° (upside down). The photographs were taken for knock-out mutants of **(A)** *GALT1*, **(B)** *GALT3*, (**C**) *GALT4* and (**D**) *GALT6* seedlings 7 d post transfer to the MS plates supplemented with 100 mM NaCl. Bar = 10 mm. **(E)** Analysis of root curvature in WT, *galt1*, *galt3*, *galt4*, *galt5* and *galt6* mutant plants. Vertical bars represent mean ± SE of the experimental means from at least three independent experiments (n=5), where experimental means were obtained from 15 seedlings per experiment. Asterisks indicate values significantly different from the WT (Dunnett's test, * P <0.05).

Supplemental Table 1. *GALT1*, *GALT3*, *GALT4*, and *GALT6* mutant and coding region information.

**Gene**	**Locus**	**Mutant lines**	**Location**	**Full length genomic DNA (bp)**	**Coding Region (bp)**	**Amino acids**	**Predicted Subcellular Localization**
GALT1	*At1g26810*	Salk_006871	Promoter	3327	1932	643	Golgi
		Sail_170A08	Intron				
GALT3	*At3g06440*	Salk_085633	Promoter	3133	1860	619	Golgi
		Salk_005178	Promoter				
GALT4	*At1g27120*	Salk_136251	Exon	2839	2022	673	Golgi
		Salk_131723	Exon				
GALT6	*At5g62620*	Sail_59_D08	Promoter	3196	2046	681	Golgi
		Sail_70_B02	Promoter				

Supplemental Table 2. Amino acid identity/similarity among the predicted amino acid sequences of GALT1-GALT6 and HPGT1-HPGT3 to GT31 family by MATCHER (http://mobyle.pasteur.fr/).

	**GALT1**	**GALT2**	**GALT3**	**GALT4**	**GALT5**	**GALT6**	**HPGT1**	**HPGT2**	**HPGT3**
**GALT1**	-	33.7	45.4	37.1	35.7	39.3	23.5	21.9	23.2
**GALT2**	51.5	-	38.9	56.4	57.1	57.9	21.6	25.5	23.5
**GALT3**	61.3	60.2	-	39.5	39.5	41.9	23.5	23.6	25.0
**GALT4**	56.1	73.2	61.4	-	68.0	60.2	23.6	20.8	24.8
**GALT5**	53.1	74.9	61.7	82.0	-	73.1	23.4	21.5	24.3
**GALT6**	56.1	74.1	64.0	73.6	82.5	-	22.6	20.0	21.9
**HPGT1**	43.6	42.7	41.6	42.7	43.8	45.7	-	41.9	43.2
**HPGT2**	43.3	49.1	44.9	42.5	43.7	46.7	64.1	-	82.4
**HPGT3**	46.3	45.9	45.8	48.6	46.7	44.8	65.2	91.6	-

Identity is displayed in the upper-half and similarity in the lower half of the table. Pair-wise analysis was done using Clustal W with a gap opening penalty of 14 and a gap extension penalty of 4 using EBLOSUM62 matrix.

Supplemental Table 3. List of candidate AGP specific glycosyltranferases and Arabidopsis AGPs coexpressed with *GALT2* and *GALT5* as query genes using the Gene CAT coexpression tool.

Pearson Correlation Coefficient	Gene ID (Trivial name)	Predicted Function
0.59084	*At4g21060 (GALT2)*	galactosyltransferase family protein, contains Pfam profile: PF01762 galactosyltransferase
0.59083	*At1g74800 (GALT5)*	galactosyltransferase family protein, contains Pfam profile: PF01762 galactosyltransferase
0.43006	*At4g32120 (HPGT1)*	galactosyltransferase family protein, contains Pfam profile: PF01762 galactosyltransferase
0.40435	*At1g32930 (GALT31A)*	galactosyltransferase family protein, contains Pfam profile: PF01762 galactosyltransferase
0.32234	*At5g53340 (HPGT2)*	galactosyltransferase family protein, contains Pfam profile: PF01762 galactosyltransferase
0.32234	*At5g53340 (HPGT3)*	galactosyltransferase family protein, contains Pfam profile: PF01762 galactosyltransferase
0.32374	*At2g35620*	leucine-rich repeat transmembrane protein kinase
0.27977	*At5g39990 (GlcAT14a)*	glycosyltransferase family 14 protein / core-2/I-branching enzyme family protein, contains Pfam profile: PF02485 Core-2/I-Branching enzyme
0.26253	*At2g37585 (GlcAT14b)*	glycosyltransferase family 14 protein / core-2/I-branching enzyme family protein, contains Pfam profile: PF02485 Core-2/I-Branching enzyme
0.24682	*At1g27120 (GALT4)*	galactosyltransferase family protein, contains Pfam profile: PF01762 galactosyltransferase
0.23551	*At3g61640*	AGP20
0.20469	*At2g14890*	AGP9
0.17328	*At2g25300*	galactosyltransferase family protein, contains Pfam profile: PF01762 galactosyltransferase
0.17184	*At5g15050 (GlcAT14c)*	glycosyltransferase family 14 protein / core-2/I-branching enzyme family protein, contains Pfam profile: PF02485 Core-2/I-Branching enzyme
0.16708	*At3g57690*	AGP23
0.13205	*At3g20865*	AGP40
0.13140	*At3g01700*	AGP11

0.10589	*At1g70630*	fucosyltransferase, putative (FUT4)
0.09032	*At1g24520*	AGP1
0.08353	*At3g06440 (GALT3)*	galactosyltransferase family protein, contains Pfam profile: PF01762 galactosyltransferase
0.07137	*At4g26320*	AGP13
0.06124	*At5g40730*	AGP24
0.05883	*At1g14080*	fucosyltransferase, putative (FUT6)
0.04499	*At5g10430*	AGP4
0.03263	*At5g56540*	AGP14

Supplemental Table 4. Subcellular distribution of Hyp-GALT activity obtained from GALT1-GALT6 transiently expressed in *N. tabacum*.

**Transgene**	**Supernatant**	**Total microsomes**	**Golgi microsomes**
None	0.82±0.02	8.14± 0.07	9.20± 0.50
GALT1	0.75±0.15	8.25± 0.02	8.95± 0.45
GALT2	0.69±0.07	9.94± 0.05[a]	14.49± 0.61[b]
GALT3	0.86± 0.07	9.85± 0.02[a]	14.46± 0.42[b]
GALT4	0.68± 0.01	9.20± 0.01[a]	13.24± 0.56[b]
GALT5	0.73± 0.02	10.39± 0.05[a]	15.27± 0.65[b]
GALT6	0.78+ 0.01	10.10± 0.07[a]	14.96± 0.71[b]

Experiments were performed using duplicate samples, and the data represent mean ± SD from three independent experiments. Letters indicate mean values significantly different from the WT (Dunnett's test, [a] $P < 0.05$; [b] $P < 0.01$).

Supplemental Table 5. List of AGP-specific glycosyltransferases and AGPs with their respective mutant phenotypes.

Gene Identifier	Function/GT Family	Mutants	Mutant Phenotype	References
At4g21060 (*GALT2*)<br>*At1g74800* *(GALT5)*	hydroxyproline-*O*-β-galactosyltransferase/ GT-31	*galt2-1* (SALK_117233)<br>*galt2-2* (SALK_141126)<br>*galt5-1* (SALK_105404)<br>*galt5-2* (SALK_115741)	Reduced root growth under salt stress, radial swelling of root tips, reduced seed mucilage adherence	[15]<br>[16]
At5g53340 (HPGT1)<br>*At4g32120 (HPGT2)*<br>*At2g25300 (HPGT3)*	hydroxyproline-*O*-β-galactosyltransferase/ GT-31	*hpgt1-1* *(*SALK_007547)<br>*hpgt2-1* (SALK_070368)<br>*hpgt3-1* (SALK_009405)	longer lateral roots, longer root hairs, radial expansion of the cells in the root tip, small leaves, shorter inflorescence stems, reduced fertility and shorter siliques	[17]
At1g77810	β-1,3-galactosyltransferase/ GT-31	Not reported	-	[18]
At5g39990 (GlcAT14A)<br>*At5g15050 (GlcAT14B)*<br>*At2g37585 (GlcAT14C)*	β-1,6-glucuronosyltransferas/ GT-14	*glcat14a-1 (SALK_06433)*<br>*glcat14a-2 (SALK_043905)*<br>Not reported<br>Not reported	Enhanced cell elongation in seedlings<br>-<br>-	[21], [22]
At2g15390 (FUT4)<br>*At1g14080 (FUT6)*	α-1,2-fucosyltransferase/ GT-37	*fut4-1 (SAIL_284_B)*<br>*fut4-2 (SALK_12530)*<br>*fut6-1 (SALK_0783)*<br>*fut6-2 (SALK_09950)*	Reduced root growth under salt stress	[12]-[14]
GALT31A (At1g32930)	β-1,6-galactosyltransferase/ GT-31	*galt31A (FLAG_379B06)*	Embryo lethal mutant	[19]
At1g08280	β-1,6-galactosyltransferase/ GT-29	Not reported	-	[20]

At1g70630 (RAY1)	β-arabinofuranosyl transferase/GT-77	*ray1-1 (SALK_053158)* *ray1-2 (GABI_001C09)*	Reduced root growth and reduced rosette size and inflorescence	[23]
At3g46550 (FLA4)	Fasciclin-like AGP	*sos5-1* (point mutation) *sos5-2 (SALK_125874)*	Shorter siliques. fewer seeds, abnormal cell expansion in salt, abnormal seed coat mucilage	[37], [69]
At2g24450 (FLA3)	Fasciclin-like AGPs	*fla3 (SALK_016582)*	Development of male reproductive organs and pollen grains	[91]
At5g55730 (FLA1)	Fasciclin-like AGPs	*fla1-1* (stock *CS01810*, Wassilewskija-2 *fla1-2 (SALK_058964)*	Shoot regeneration	[92]
At5g03170 (FLA11) *At5g60490 (FLA12)*	Fasciclin-like AGPs	*fla11 (SALK_046976)* *fla12* *SM.15162*	Synthesis of secondary cell wall	[93]
At5g14380 (AGP6)	Classical AGP	RNAi and ami RNA lines	Growth and development of pollen grain and pollen tube	[57]
At3g01700 (AGP11)	Classical AGP	RNAi and ami RNA lines	Growth and development of pollen grain and pollen tube	[60]
At2g23130 (AGP17)	Lys-rich classical	*agp17 (SALK_101062)*	Influence *Agro-bacterium* binding	[94], [95]
At4g37450 (AGP18)	Lys-rich classical	*agp18 (SALK_117268)*	Mediated megaspore selection, reduced hypocotyl cell length, compromised fertility	[62], [94], [96]
At1g68725 (AGP19)	Lys-rich classical	*agp19 (SALK_038728)*	Reduced hypocotyl cell length, compromised fertility	[94]
At2g33790 (AGP30)	Chimeric AGP	*agp30* (transposon insertion)	Root regeneration and seed germination	[97]

Supplemental Table 6. List of primers used in this study.

Purpose	Forward	Reverse
**Cloning**		
GALT1	CACCATGCATCATCATCATCATCACAAG AGATTTTATGGAGGGCTTC	TTACCATTCGCGGCAGCAAA
GALT3	CACCATGCATCATCATCATCATCACAAG CAATTCATGTCAGTGGT	TTATTCGCAGCAAATAGATTGGTTC
GALT4	CACCATGCATCATCATCATCATCACAAG AAGTCTAAACTCGATAATTC	TCATCTCATGTTGCAGCATTG
GALT6	CACCATGCATCATCATCATCATCACAGG AAGCCCAAGTTGTCA	TCATCTCATGTTGCAGCACTG
**Subcellular localization**		
GALT3	CAGGACGTCTAGATGAAGCAATTCATGT CAGTGGTGAGATTC	CATGACCGTCGACTTTTCGCAGCAAATA GATTGGTTCTC
GALT4	CAGGACGTCTAGATGAAGAAGTCTAAA CTCGAT	CATGACCGTCGACTTTCTCATGTTGCAG CATTG
GALT6	CAGGACGTCTAGATGAGGCCCAAGTTG TCA	CATGACCGTCGACTTTCTCATGTTGCAG CACTG
**Screening for T-DNA**		
galt1-1	TTTTTCACAGCCGAAAATCAC	TTGGGAACTTGTTTTTACCCC
galt1-2	GAGTTCCAGTAGCCAGGGAAG	TTCGAATAGGTTGAGAGTCGG
galt3-1	AGGCAAATGGAATAACTTGGC	TGGGGTTACTTCGCTTACATG
galt3-2	ACTGGTTTCTTCGTGGTTGTG	TGAATTGGTGCAGAAAGGATC
galt4-1	GATTAAACCCGAATCGAGTCC	TTTGAACTTGGAATTTGGTCC
galt4-2	GACTTCCTTTCTTGCATGCTG	GGACTCGATTCGGGTTTAATC
galt6-1	AGAACACGAGTTTGTCCCATG	TTTTGGTCGATTTGCTTAACC
galt6-2	GATGCAAAGGTGTCACACATG	CTCGAGTTTTGACAACTTGGG
LBa1.3	ATTTTGCCGATTTCGGAAC	
LB3	TAGCATCTGAATTTCATAACCAAT CTCGATACAC	
**RT-PCR**		
GALT1	CATCTTCGGGACAGAGGTTG	AAACCAAACGCTCTCTTTGCTGC
GALT2	TCTTTGTTGCACTTAATCCAAGAAG	TGTGGTCGACCTTTCAACAAATTAT
GALT3	GTTGACTACTATGGTTTACTTAGCTTG	TTATTCGCAGCAAATAGATTGGTTCTC
GALT4	CTTTGTGGCATTGCATGCAAGAAAG	CCATCTTGAATAATCTTAATCGCTTTTG

GALT5	TATGTGAACACGGAGCTCTTGCATTC	TCCATCTTGAACAGCCGTAATTTATGTCT
GALT6	GCAATTTGCGAGTACGGGGCTCATCAG	CGCCGTCAAGTAATTCTCTATGC
UBQ10	TCGACCCTTCACTTGGTGT	ATAAGCTGGTGTTGACAGGCA
**qRT-PCR**		
GALT1	AGGAAACCTAAAGATGTTTAAGCTG	GCAGCAAAGAGAGCGTTTGGTTT
GALT2	CATAAGCTTAGGCTATTCAAGATGG	GGTCGACCTTTCAACAAATTAT
GALT3	AGCGAAATTT GTGGTGAAGG	TTATTCGCAGCAAATAGATTGGTT
GALT4	CCAGAAGAGTATTATCCTCCATATG	GCTTATCCCACATGCAAATCAT
GALT5	ACATAAATTACGGCTGTTCAAGATGGA	TCTTAAGAGCTTATCCCATAAGCATA
GALT6	ACACAAATTAAGGATGTTCAAAATGG	TGCCTGTCAACACCAGCTTAT

Supplemental Figure 1

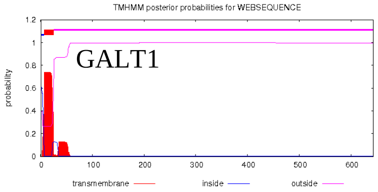

WEBSEQUENCE Number of predicted TMHs: 1
WEBSEQUENCE Exp number of AAs in TMHs: 16.84169
WEBSEQUENCE Exp number, first 60 AAs: 16.77893
WEBSEQUENCE Total prob of N-in: 0.60734 (N-term signal sequence)

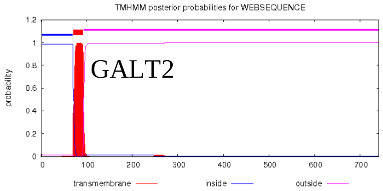

WEBSEQUENCE Number of predicted TMHs: 1
WEBSEQUENCE Exp number of AAs in TMHs: 22.44547
WEBSEQUENCE Exp number, first 60 AAs: 0.02139
WEBSEQUENCE Total prob of N-in: 0.98531 (N-term signal sequence)

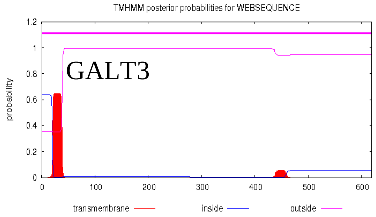

WEBSEQUENCE Number of predicted TMHs: 0
WEBSEQUENCE Exp number of AAs in TMHs: 13.20133
WEBSEQUENCE Exp number, first 60 AAs: 11.93726
WEBSEQUENCE Total prob of N-in 0.64257

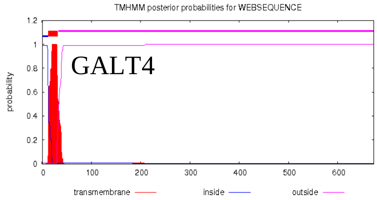

WEBSEQUENCE Number of predicted TMHs: 1
WEBSEQUENCE Exp number of AAs in TMHs: 20.75225
WEBSEQUENCE Exp number, first 60 AAs: 20.56502
WEBSEQUENCE Total prob of N-in: 0.99144 (N-term signal sequence)

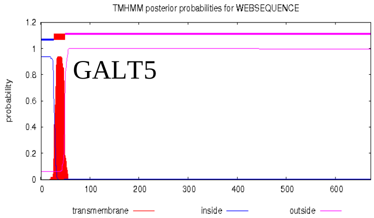

WEBSEQUENCE Number of predicted TMHs: 1
WEBSEQUENCE Exp number of AAs in TMHs: 19.95121
WEBSEQUENCE Exp number, first 60 AAs: 19.86042
WEBSEQUENCE Total prob of N-in: 0.93780 (N-term signal sequence)

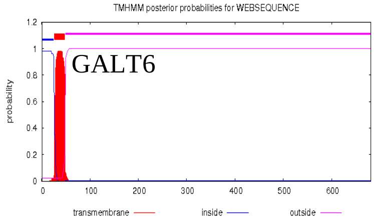

WEBSEQUENCE Number of predicted TMHs: 1
WEBSEQUENCE Exp number of AAs in TMHs: 21.23239
WEBSEQUENCE Exp number, first 60 AAs: 21.22707
WEBSEQUENCE Total prob of N-in: 0.97955 (N-term signal sequence)

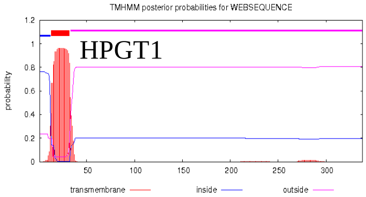

WEBSEQUENCE Number of predicted TMHs: 1
WEBSEQUENCE Exp number of AAs in TMHs: 20.29915
WEBSEQUENCE Exp number, first 60 AAs: 20.06079
WEBSEQUENCE Total prob of N-in: 0.76285 (N-term signal sequence)

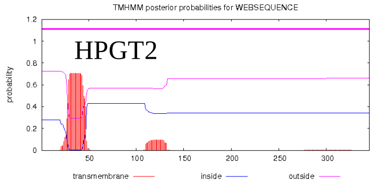

WEBSEQUENCE Number of predicted TMHs: 0
WEBSEQUENCE Exp number of AAs in TMHs: 15.80752
WEBSEQUENCE Exp number, first 60 AAs: 13.76952
WEBSEQUENCE Total prob of N-in: 0.27752

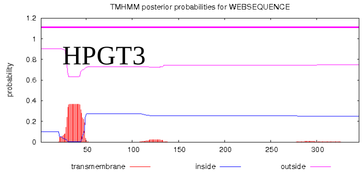

WEBSEQUENCE Number of predicted TMHs: 0
WEBSEQUENCE Exp number of AAs in TMHs: 7.66106
WEBSEQUENCE Exp number, first 60 AAs: 7.14472
WEBSEQUENCE Total prob of N-in: 0.09766

Supplemental Figure 2

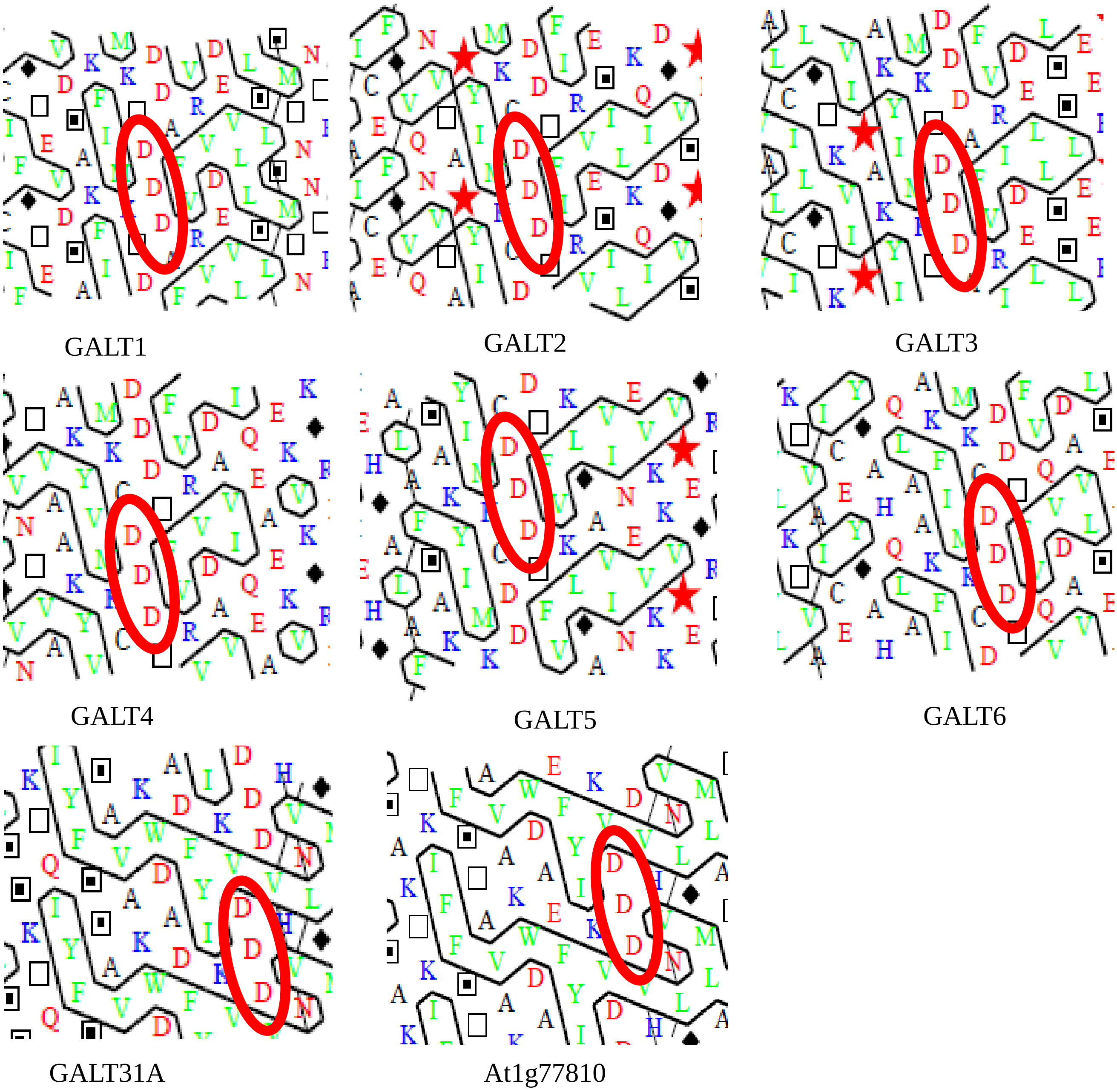

Supplemental Figure 3

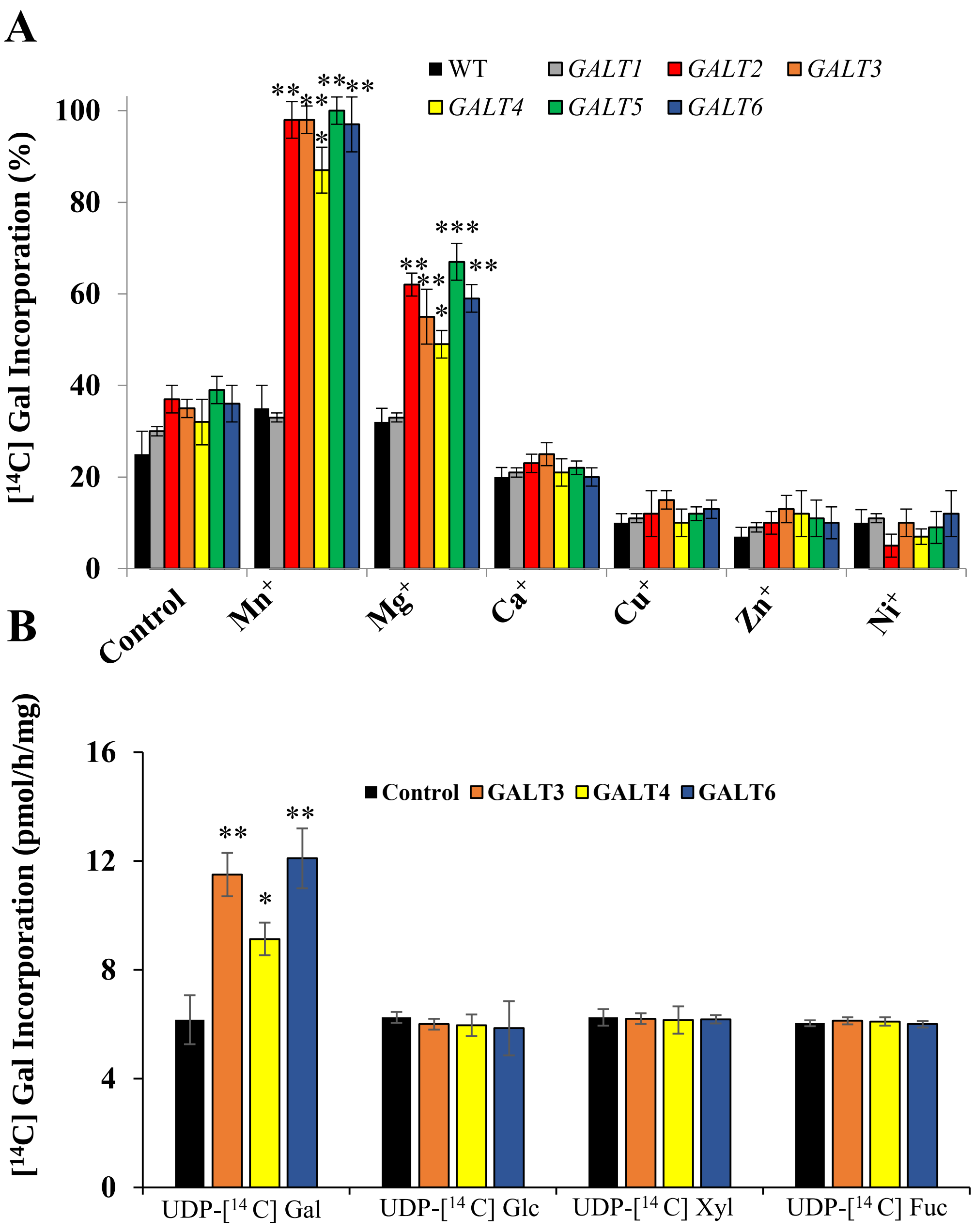

Supplemental Figure 4

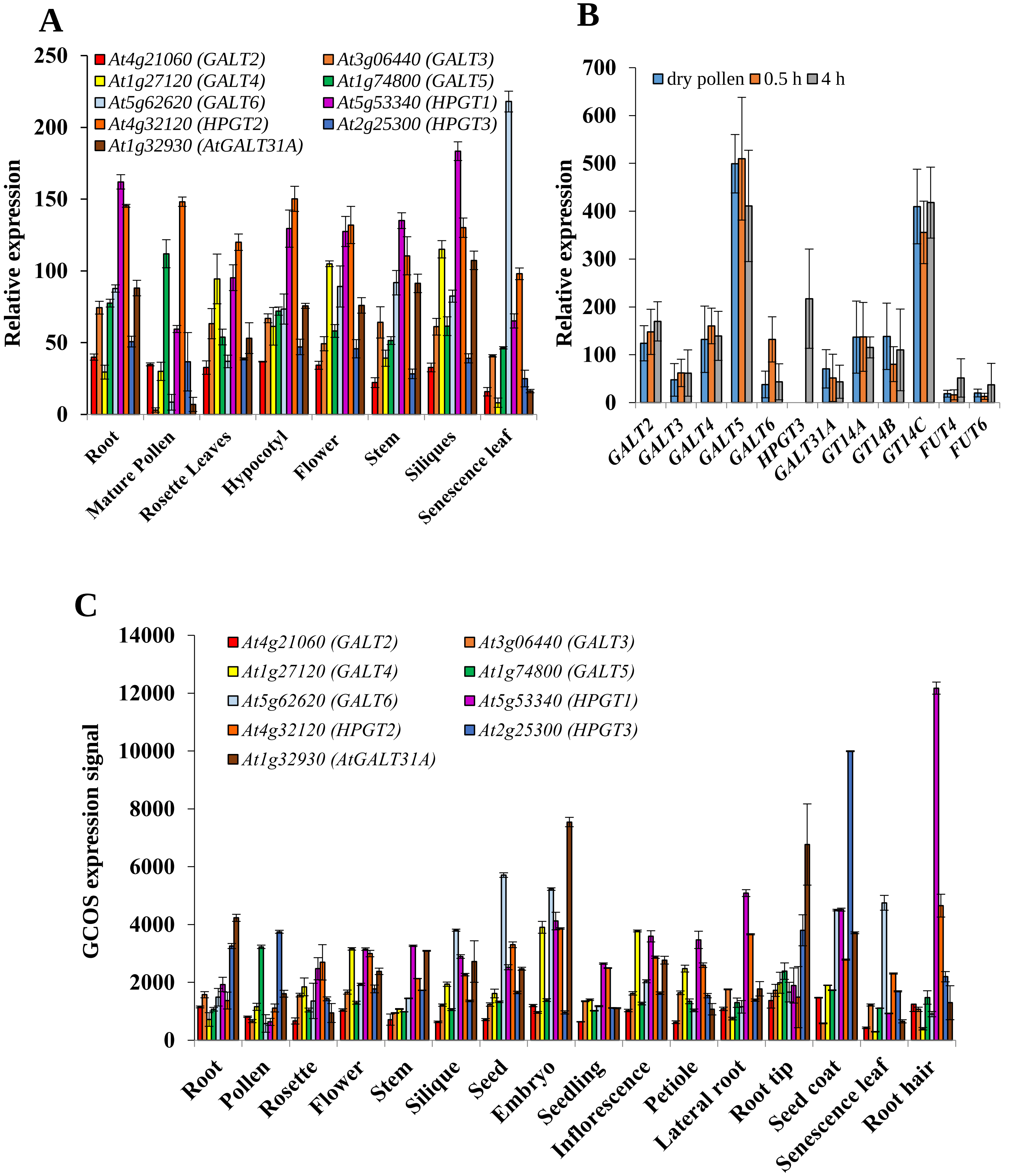

Supplemental Figure 5

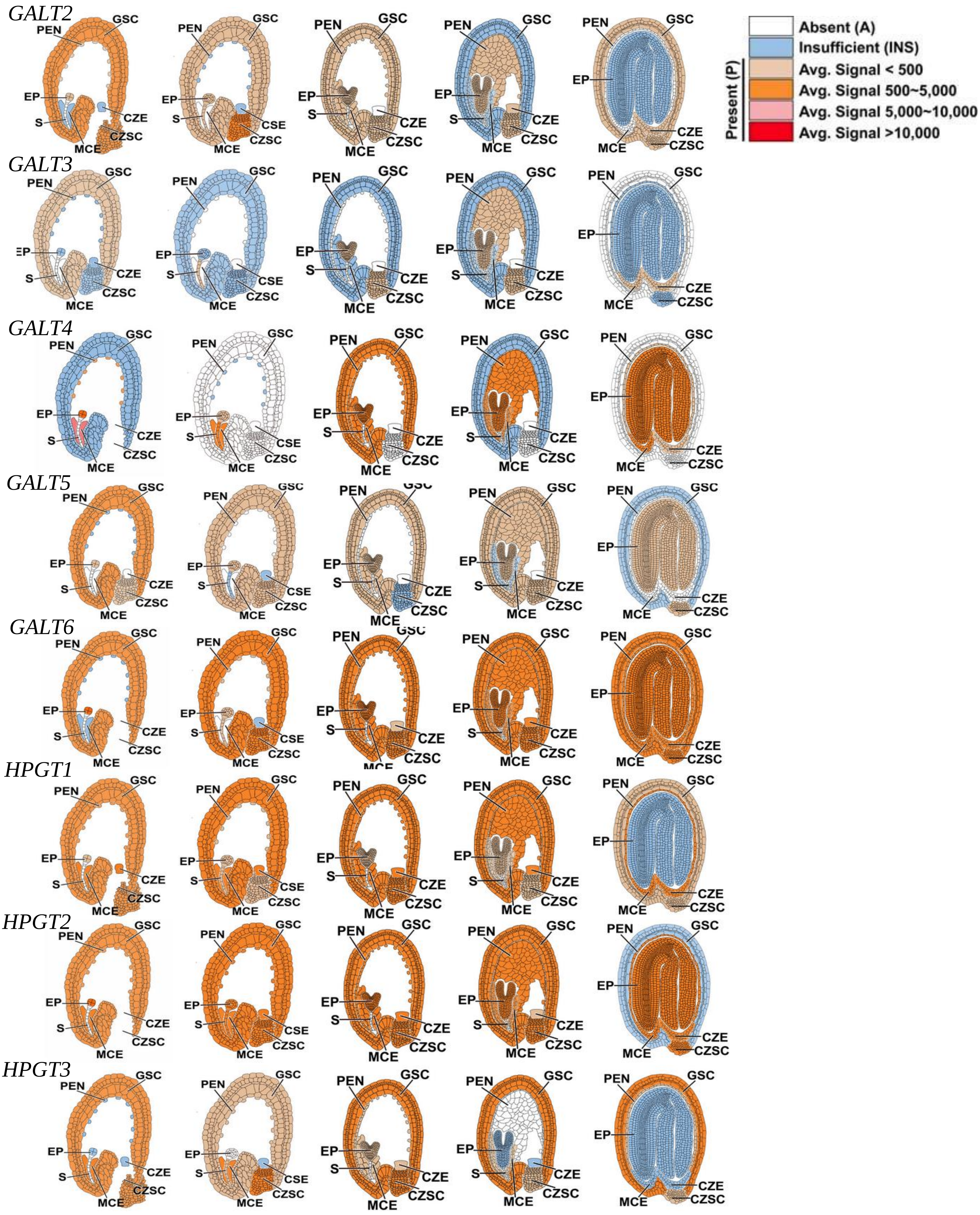

Supplemental Figure 6

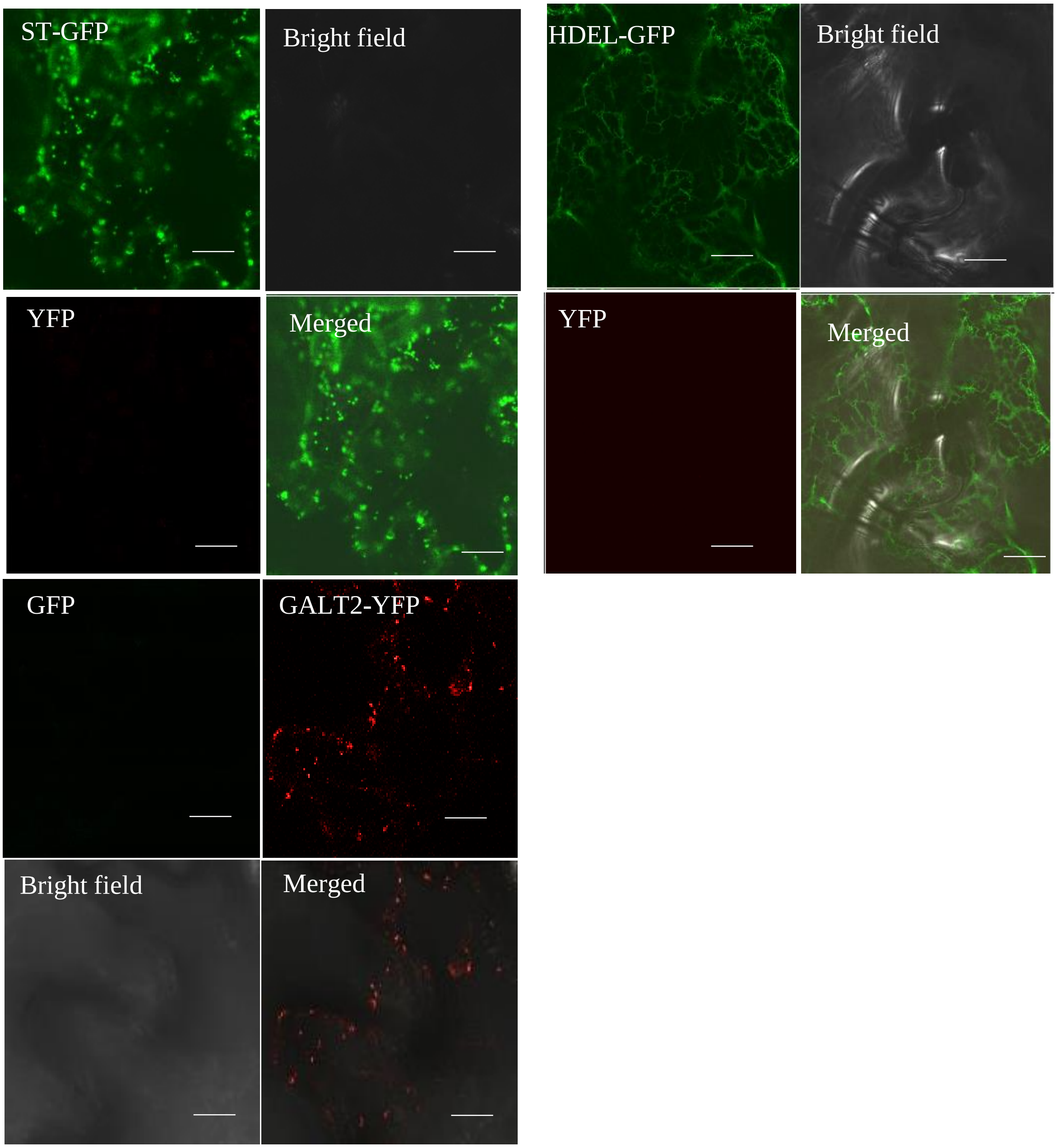

Supplemental Figure 7

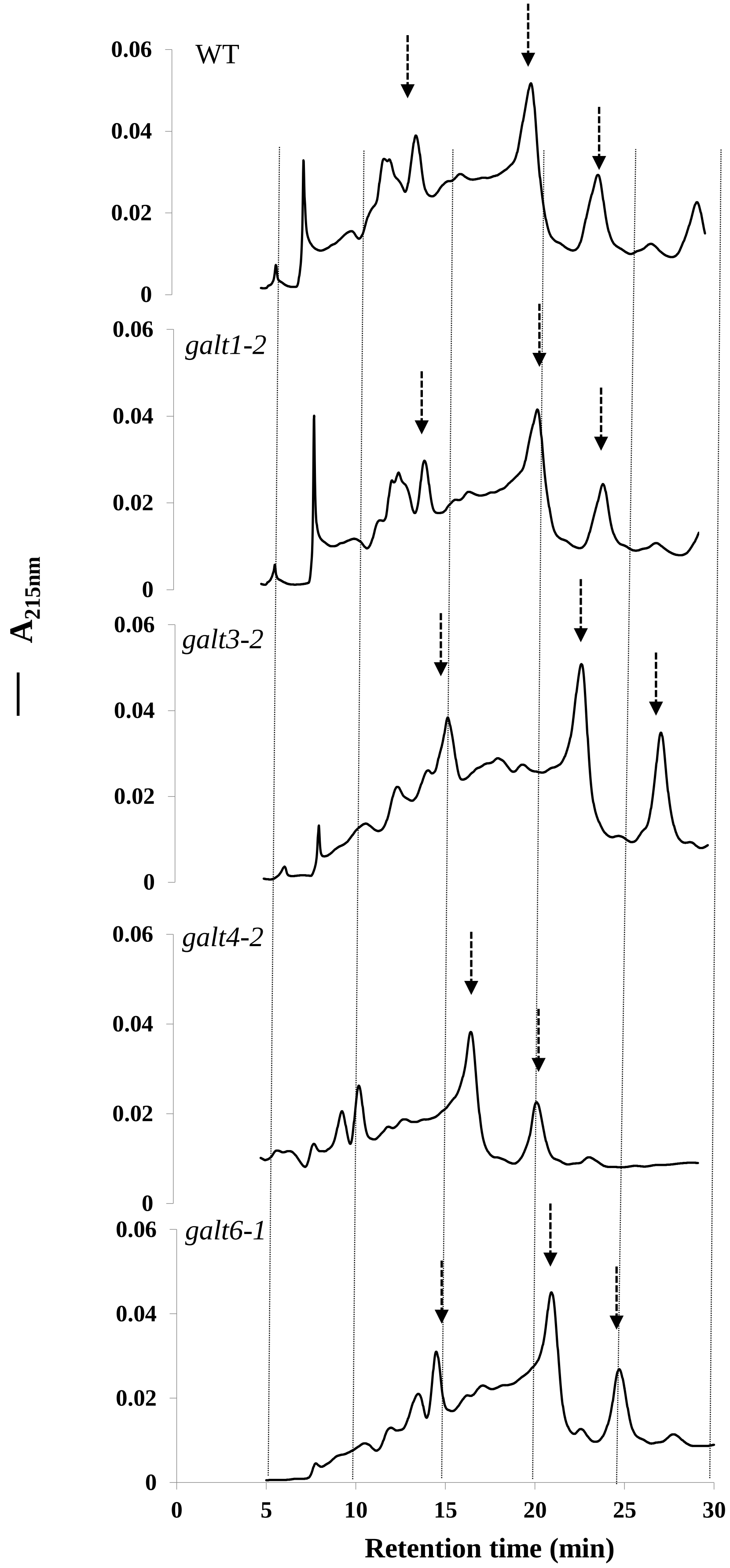

Supplemental Figure 8

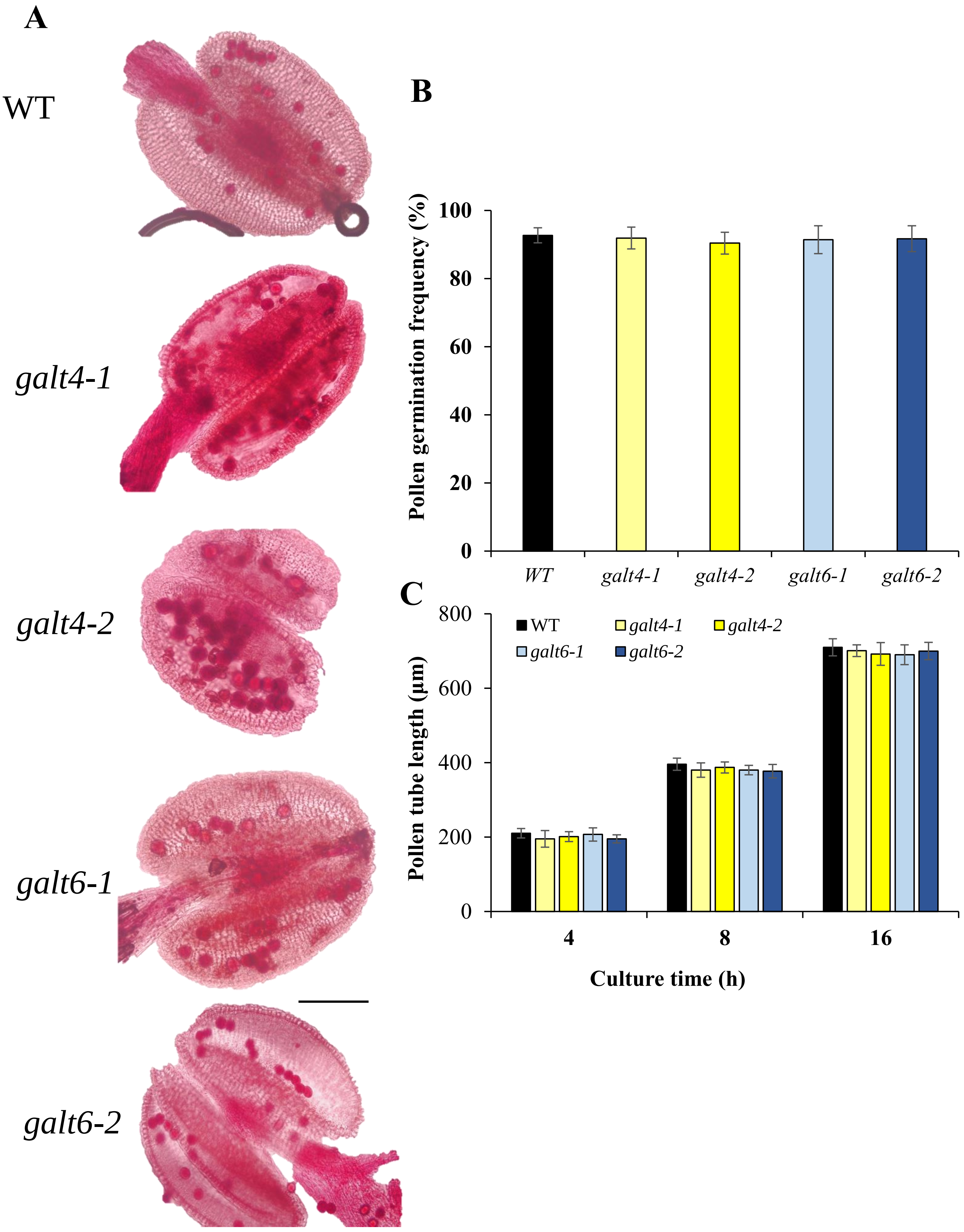

Supplemental Figure 9

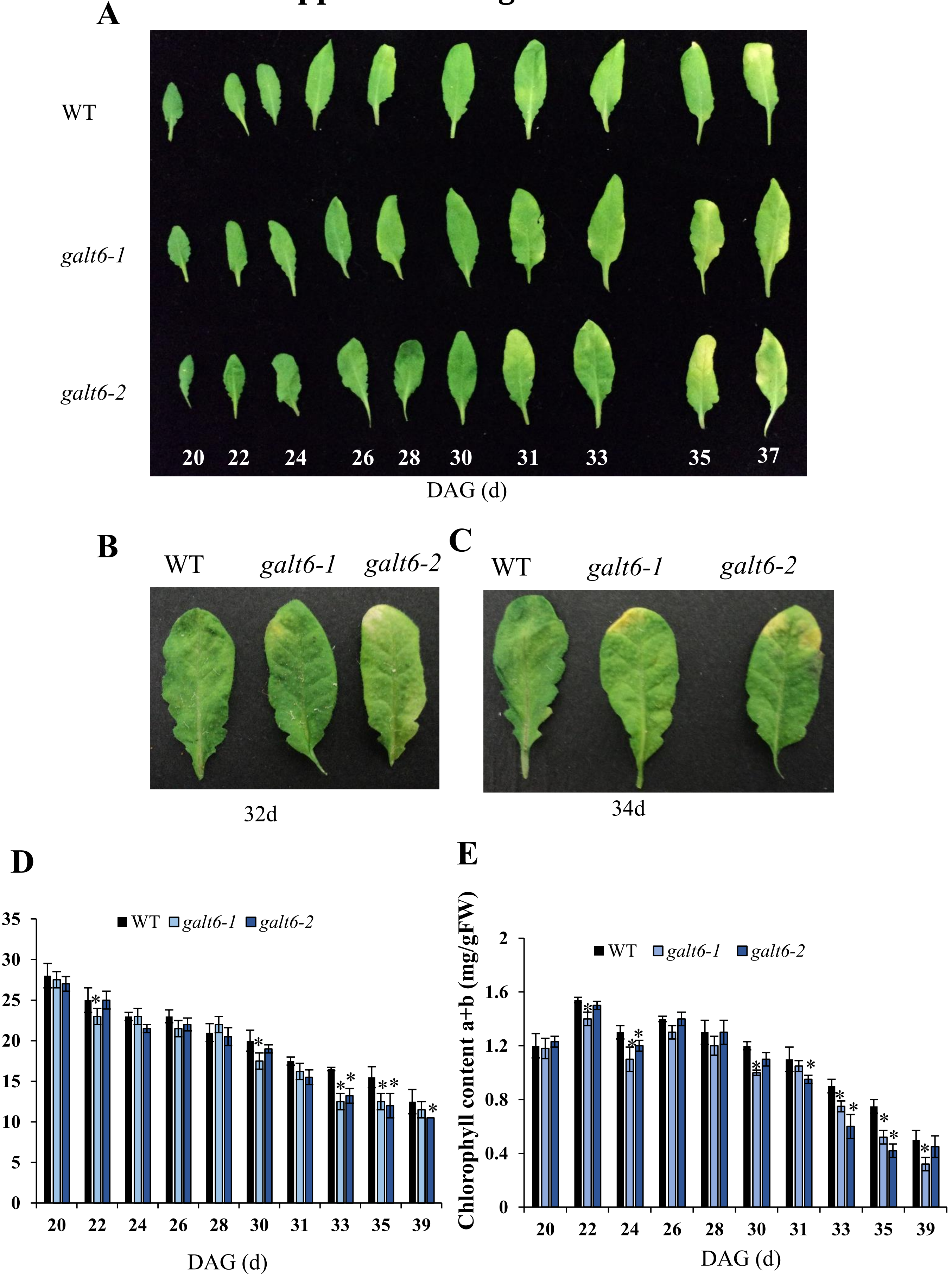

Supplemental Figure 10

A

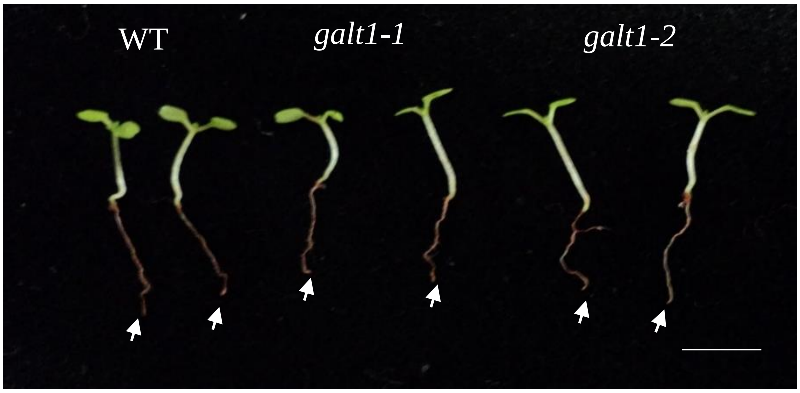

B

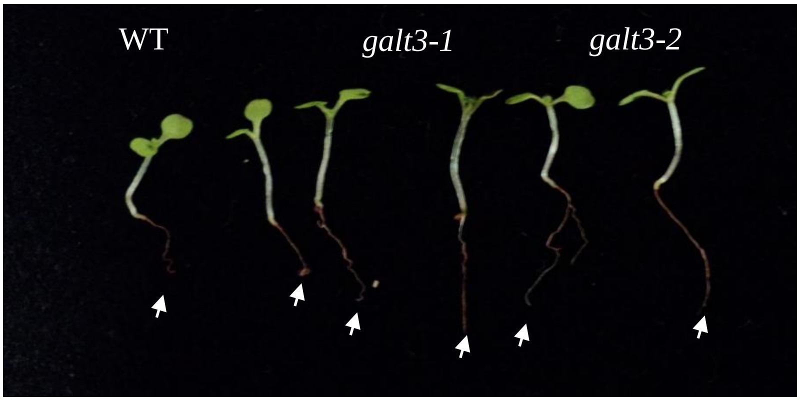

C

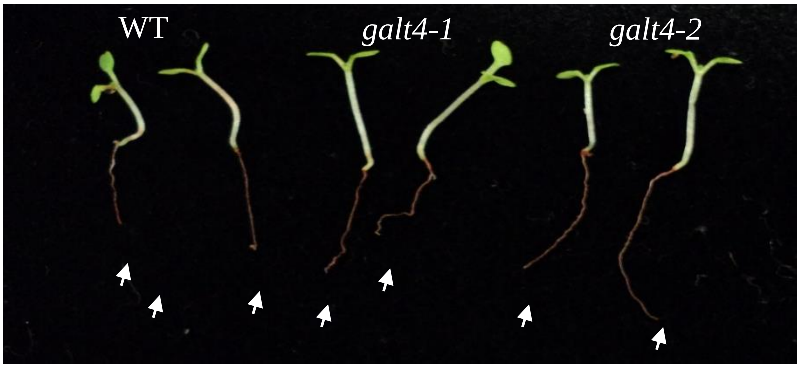

D

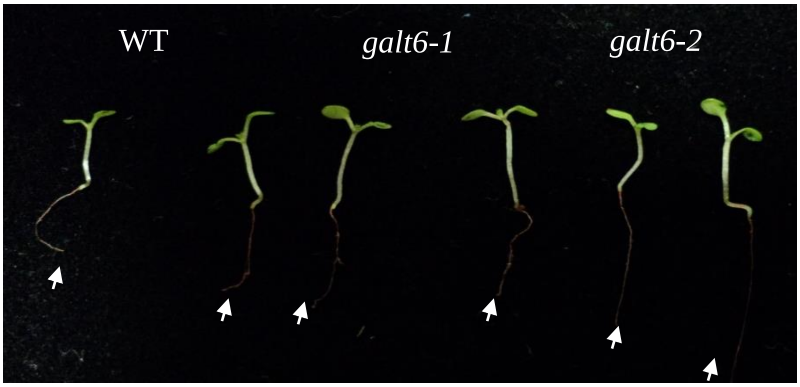

Supplemental Figure 11

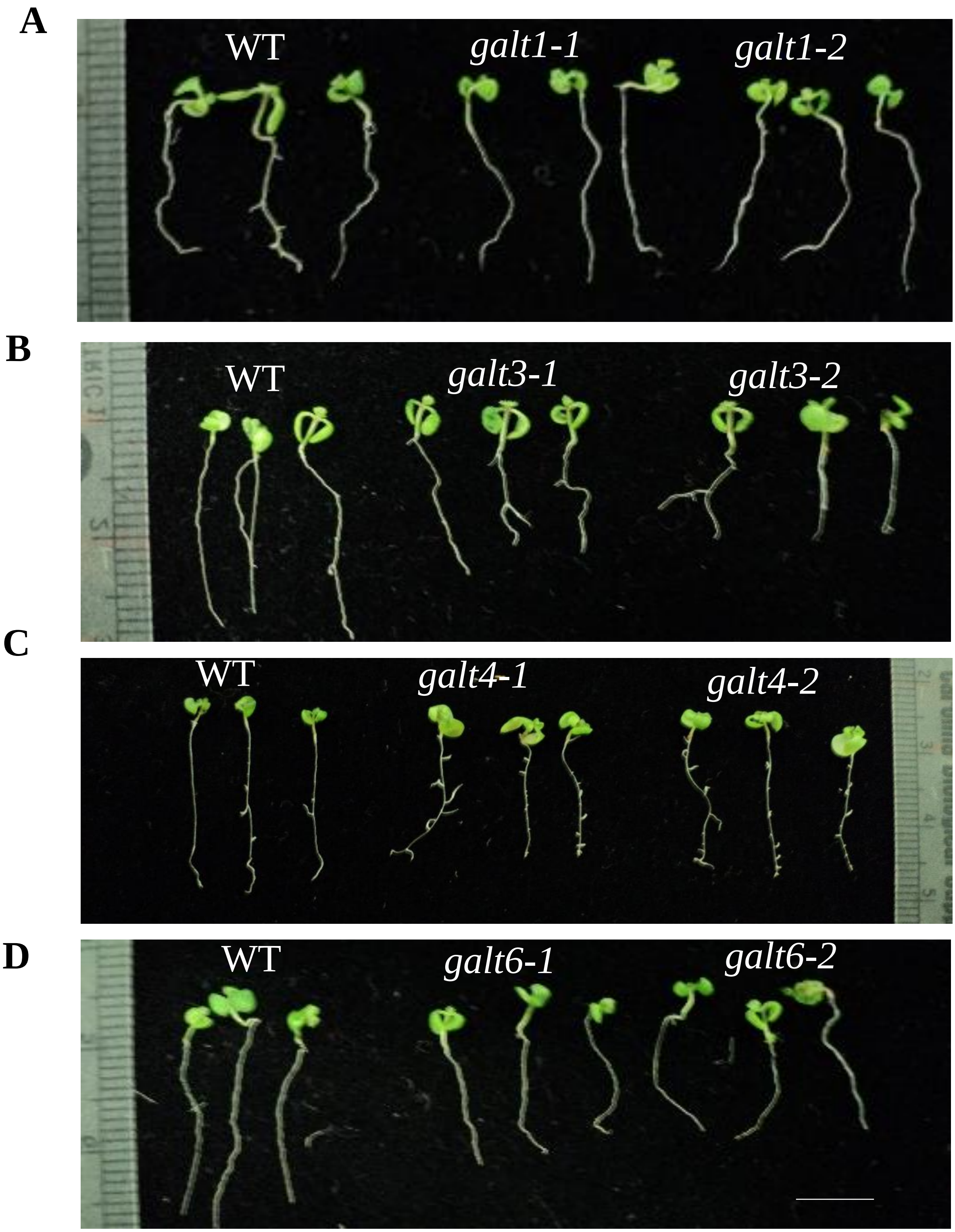

Supplemental Figure 12

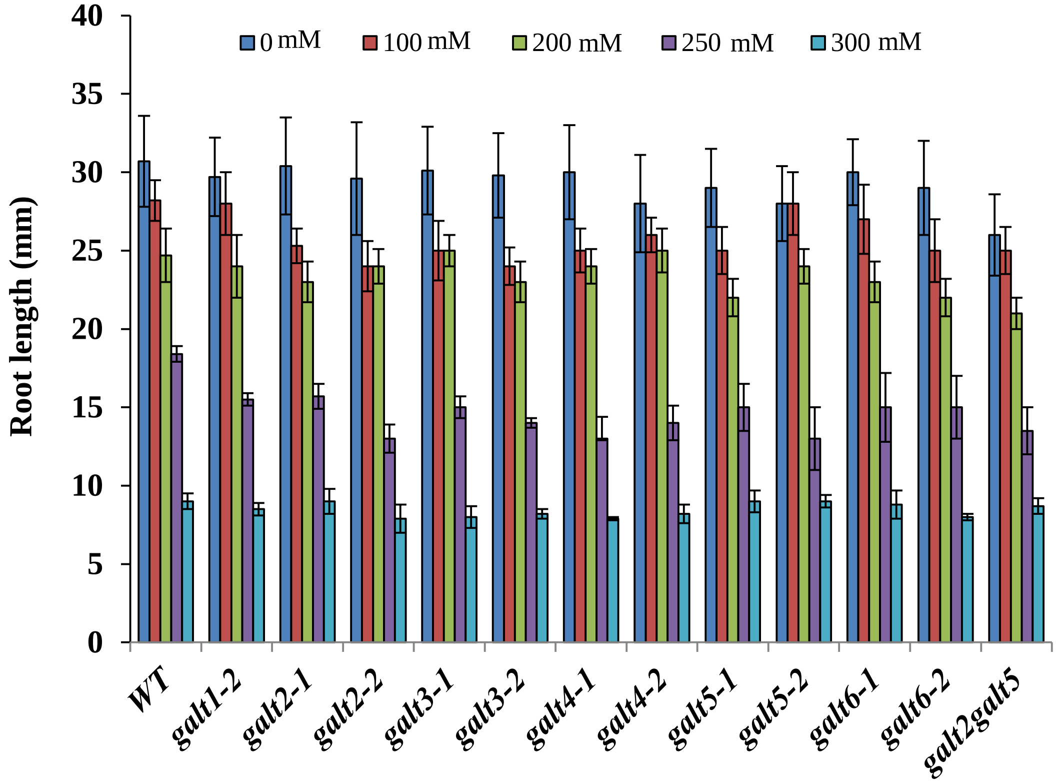

Supplemental Figure 13

A

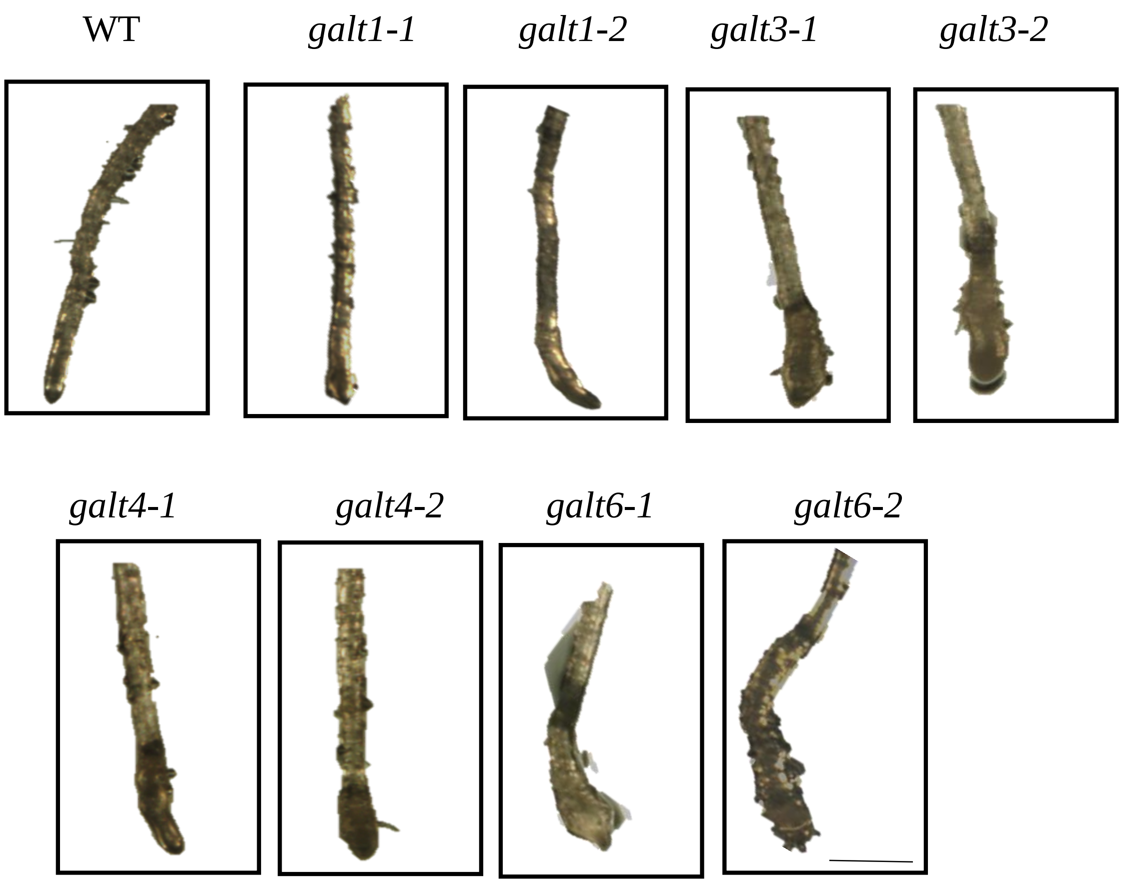

B

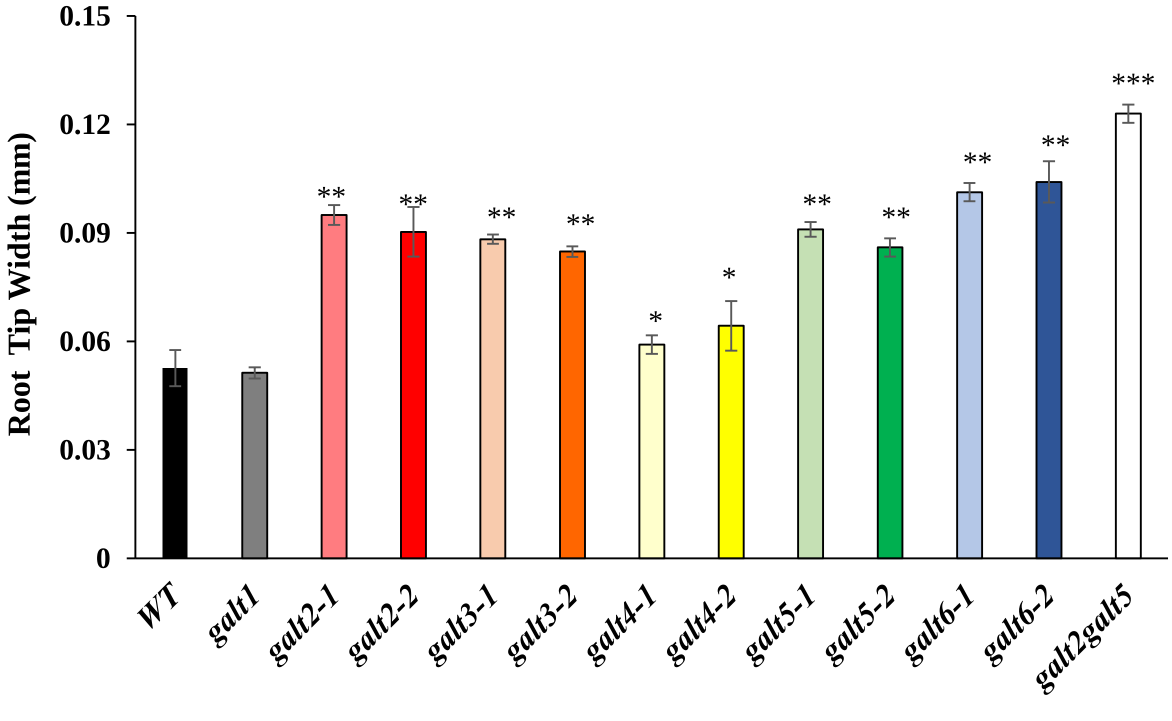

Supplemental Figure 14

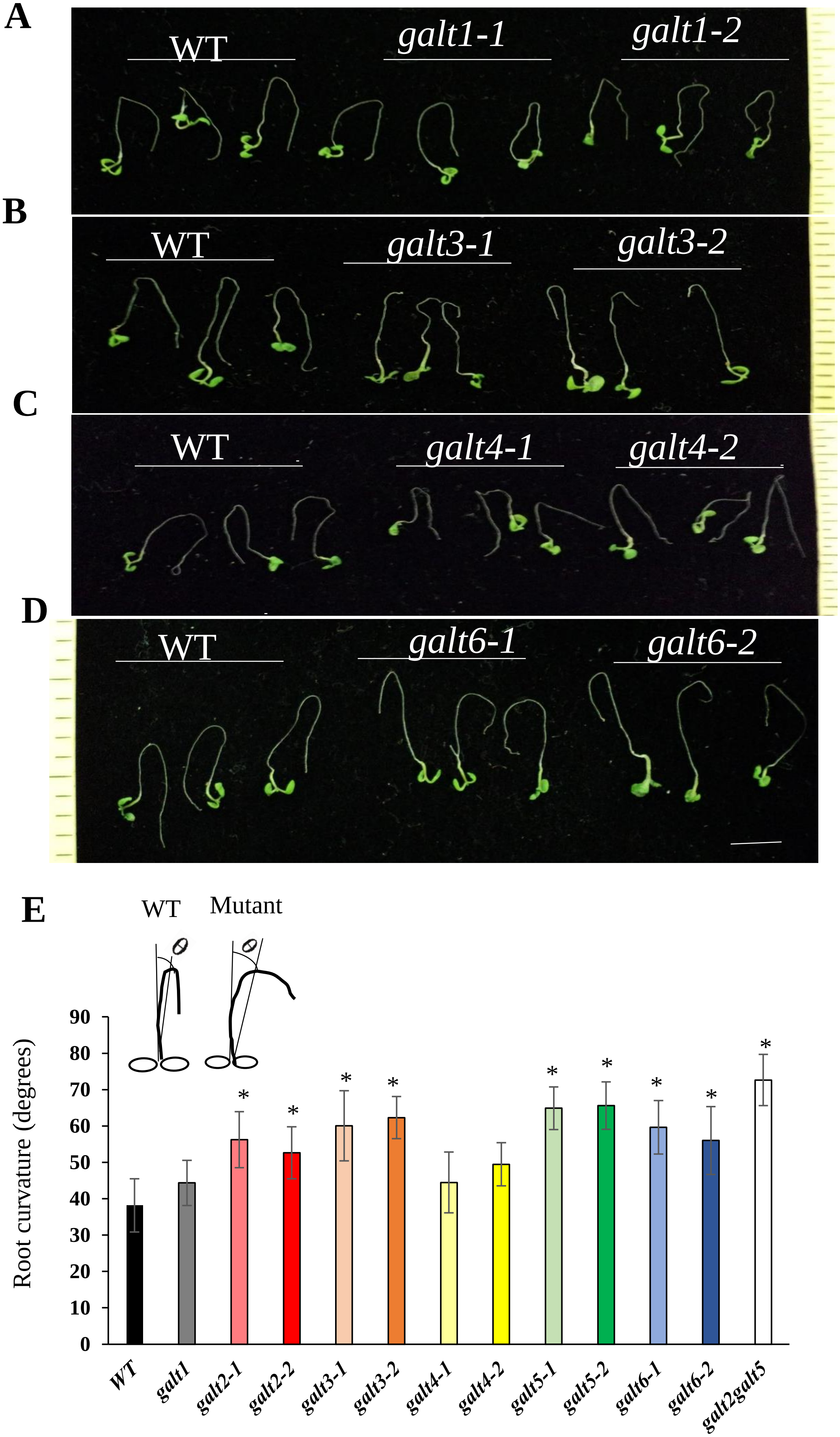

Supplement: Additional file 1: — Six supplemental tables, 14 supplemental figures and their corresponding legends. Table S1. GALT1, GALT3, GALT4, and GALT6 mutant and coding region information. Table S2. Amino acid identity/similarity among the predicted amino acid sequences of GALT1-GALT6 and HPGT1-HPGT3 to GT31 family by MATCHER (http://mobyle.pasteur.fr/). Table S3. List of candidate AGP specific glycosyltranferases and Arabidopsis AGPs coexpressed with GALT2 and GALT5 as query genes using the Gene CAT coexpression tool. Table S4. Subcellular distribution of Hyp-GALT activity obtained from GALT1-GALT6 transiently expressed in N. tabacum. Table S5. List of AGP-specific glycosyltransferases and AGPs with their respective mutant phenotypes [15–22, 37, 57, 60, 62, 69, 91–97]. Table S6. List of primers used in this study. Figure S1. Predicted transmembrane regions of GALT1-GALT6 and HPGT1-HPGT3. Figure S2. Hydrophobic cluster (HCA) analysis of GALTs showing the DXD motif within a pocket of hydrophobic amino acids. Figure S3. Biochemical characterization of Hyp-GALT activity. Figure S4. Gene expression profile of Hyp-GALTs and GALT1 in different organs/tissues. Figure S5. Transcript levels of Hyp-GALTs in the developing seed coat depicted by http://seedgenenetwork.net/ [36]. Figure S6. Single infiltration in tobacco epidermal cells. Tobacco leaves were infiltrated with either with ST-GFP, HDELGFP, or GALT2-YFP as indicated. Size bar = 10 μm. Figure S7. RP-HPLC profiles of AGPs extracted from WT and single galt mutants. Figure S8. Pollen viability, pollen germination frequency and pollen tube growth of galt4 and galt6 mutants. Figure S9. Age-dependent leaf senescence phenotype of galt6-1 and galt6-2 mutant plants. Figure S10. Representative images of WT and galt roots treated with 50 μM β -Gal-Yariv reagent. Figure S11. Representative images of WT, galt1, galt3, galt4, and galt6 plants after 14 days of growth on MS plates supplemented with 100 mM NaCl. Figure S12. The galt mutants are insensitive to ma [file 12870_2015_670_MOESM1_ESM.pdf]
